# Cardiovascular safety of biologic therapies in patients with severe asthma: a nationwide cohort study in Belgium

**DOI:** 10.1016/j.lanepe.2025.101420

**Published:** 2025-08-06

**Authors:** Frauke Van Vaerenbergh, Delphine Vauterin, Maxim Grymonprez, Lowie E.G.W. Vanfleteren, Guy Brusselle, Lies Lahousse

**Affiliations:** a Department of Bioanalysis, Faculty of Pharmaceutical Sciences, Ghent University, Ottergemsesteenweg 460, Ghent, 9000, Belgium; b Department of Cardiology, Ghent University Hospital, Ghent University, Corneel Heymanslaan 10, Ghent, Belgium; c COPD Center, Department of Respiratory Medicine and Allergology, Sahlgrenska University Hospital, Gothenburg, Sweden; d Department of Internal Medicine and Clinical Nutrition, Institute of Medicine, Sahlgrenska Academy, University of Gothenburg, Gothenburg, Sweden; e Department of Respiratory Medicine, Ghent University Hospital, Ghent, Belgium; f Department of Epidemiology, Erasmus Medical Center, Dr. Molewaterplein 40, Rotterdam, the Netherlands; g Department of Respiratory Medicine, Erasmus Medical Center, Dr. Molewaterplein 40, Rotterdam, the Netherlands

**Keywords:** Monoclonal antibody, Asthma, Biological, Cardiovascular events, Mortality, Anti-IgE therapy, Anti-IL5 therapy

## Abstract

**Background:**

In last decades, biologic therapies have been approved for severe allergic and/or eosinophilic asthma. Limited studies have investigated the effect of biologics on (acute) cardiovascular events, which have reported conflicting results. We aimed to investigate the potential cardiovascular risk of anti-immunoglobulin(Ig)-E (omalizumab) and anti-interleukin(IL)-5/IL5 receptor (IL5R) therapies (mepolizumab and benralizumab) in patients with severe asthma compared with non-biologic users.

**Methods:**

Adult asthma patients eligible for biologics were identified in Belgian nationwide data between 2017 and 2022. Inverse probability of treatment weighted Cox regression was used to investigate cardiovascular outcomes and all-cause mortality, while controlling for age, sex, obesity, smoking, comorbidities, comedication, exacerbations, and frailty.

**Findings:**

This cohort study consisted of 171,865 patients (mean age 64 years; 55% females) including 1826 (1.1%) anti-IgE users and 2398 (1.4%) anti-IL5/IL5R users. Anti-IgE exposure was associated with a significantly lower risk of mortality (aHR 0.48, 95% CI 0.40–0.58), congestive heart failure (aHR 0.79, 95% CI 0.65–0.95), peripheral artery disease (aHR 0.66, 95% CI 0.51–0.86), and stroke (aHR 0.54, 95% CI 0.36–0.81). Anti-IL5/IL5R use was associated with a significantly lower risk of mortality (aHR 0.35, 95% CI 0.29–0.42), congestive heart failure (aHR 0.63, 95% CI 0.52–0.76), arrythmia (aHR 0.78, 95% CI 0.68–0.90), and peripheral artery disease (aHR 0.69, 95% CI 0.54–0.87) compared with non-biologic users. No significant differences in the risk of myocardial infarction and pulmonary embolism were observed.

**Interpretation:**

In this nationwide observational study, biologic therapies for patients with severe asthma were associated with a significantly lower risk of all-cause mortality and specific cardiovascular diseases compared with non-biologic users.

**Funding:**

None.

**Research in context**

**Evidence before this study**

We searched PubMed for studies published between database inception and Feb 20, 2025, investigating long-term cardiovascular safety of biologic therapies in adult asthma patients. We used terms related to biologic therapies (“omalizumab”, “benralizumab”, “mepolizumab”, “reslizumab”, “dupilumab”), cardiovascular safety (“cardiovascular”), and asthma. A systematic review evaluating the safety of biologic therapies based on randomized controlled trials did not find cardiovascular-related safety issues; however, these findings were limited by enrollment of asthma patients without major cardiovascular morbidity at baseline, a restricted follow-up time and low cardiovascular event rates. A post-marketing observational cohort study, as well as pharmacovigilance studies, found an increased risk of arterial thromboembolic events in patients treated with omalizumab compared with the control group. However, other studies found no association or a protective effect of biologics on cardiovascular events.

**Added value of this study**

To the best of our knowledge, this is the first study to investigate the cardiovascular safety of anti-interleukin-5/interleukin-5R and anti-immunoglobulin-E therapy in a nationwide real-life population with a follow-up time of 495,802 person-years. We found that severe asthma patients on biologic therapies had a significantly lower risk of cardiovascular events (except for myocardial infarction and pulmonary embolism where no significant differences were found) and all-cause mortality compared with similar patients not on biologic therapies. Our results suggest that the cardiovascular protective effects of the investigated biologic therapies may go beyond reducing exacerbation rates or oral corticosteroid use.

**Implications of all the available evidence**

Our findings suggest that biologics might have cardiovascular protective effects in adult patients with severe asthma. Future research is needed to investigate whether the observed association is causal, and to unravel the underlying mechanisms, explaining the main drivers of the effect, and thereby providing further insights into the link between asthma and cardiovascular diseases.

## Introduction

Asthma, a heterogeneous obstructive respiratory disease, affects around 300 million individuals worldwide.^1^^,^^2^ The majority of asthma patients can be effectively treated with inhaled corticosteroids (ICS, with or without a second controller).^3^^,^^4^ Nevertheless, approximately one in five asthma patients are estimated to be uncontrolled, which largely affects their quality of life, hospitalizations, morbidity and mortality risk, and associated costs.^3^^,^^5^ Moreover, research has demonstrated that uncontrolled asthma is associated with an increased risk of cardiovascular (CV) disease.^6^, ^7^, ^8^, ^9^ Patients whose asthma remains uncontrolled despite high intensity treatment with good adherence, and optimal management of comorbidities and risk factors, referred to as severe asthma, are eligible for add-on biologic therapies.^1^^,^^4^

In the last decades, specific targeted therapies have been approved for severe allergic and/or eosinophilic asthma, including monoclonal antibodies targeting immunoglobulin(Ig)-E, interleukin(IL)-5, IL-5 receptor (IL5R), or IL-4 receptor alpha (IL4Rα) (blocking the signaling of both IL-4 and IL-13).^10^^,^^11^ Randomized controlled trials (RCTs) for these biologic therapies have demonstrated a reduction in exacerbation rates, an oral corticosteroid(OCS)-sparing effect, and improvements in quality of life and symptom control with a good safety profile.^1^^,^^10^ However, strict eligibility criteria are often applied in RCTs, implicating the presence of major differences in patient characteristics and comorbidities between clinical trial-eligible and real-world populations.^12^ Moreover, whereas severe asthma in adulthood is a lifelong disease, RCTs often have a limited follow-up time, increasing the need for real-world studies investigating long-term safety.

Limited studies have investigated the effect of biologics on CV events and conflicting results have been reported.^7^ A post-marketing observational cohort study of omalizumab found an elevated risk of CV and cerebrovascular (CBV) events in patients treated with omalizumab compared with the control group, possibly due to variations in asthma severity.^13^ Moreover, pharmacovigilance studies reported more CV suspected adverse drug reactions with omalizumab.^14^, ^15^, ^16^ Other studies found no associations or even a protective effect of biologics on CV events.^17^, ^18^, ^19^, ^20^

Therefore, we aimed to investigate the risk of CV events of anti-IgE and anti-IL5/IL5R therapies in adult patients with severe asthma by using data on a nationwide scale from 2017 to 2021.

## Methods

This study followed the Strengthening the Reporting of Observational Studies in Epidemiology (STROBE) reporting guideline (eTable 1).

### Source population

The source population was provided by two Belgian nationwide databases, namely the InterMutualistic Agency (IMA) database and the Minimal Hospital Dataset (MHD). The IMA centralizes all claims data from Belgian health insurance funds and manages three databases: (i) a population database containing socio-demographic characteristics (e.g., sex, age, insurance status, and date of death), (ii) a healthcare database containing data on reimbursed ambulatory and hospital care (e.g., medical procedures, inpatient medication, and other reimbursed care), and (iii) a pharmaceutical database containing outpatient medication dispensing data (e.g., dispensing date, Anatomical Therapeutic Chemical (ATC) Classification code). Since health insurance is legally mandatory in Belgium, the source population represents all legal residents with reimbursed medication or care. The MHD, collected by the Belgian Ministry of Health, aggregates hospital discharge diagnoses of every hospital admission in Belgium, coded in International Classification of Diseases (ICD) codes. The Trusted Third Party ‘eHealth’ was responsible for linking both databases using the national social security number as unique patient identifier. After applying an encrypting procedure for privacy protection, only pseudonymized data were available to the researchers on the secured IMA servers. This study was approved by the IMA and MHD database administrators, and by the ‘Social Security and Health Chamber of the Belgian Information Security Committee’ (approval code IVC/KSZG/23/008), waiving the need for individual informed consents.

### Study population

Within all adult (≥18 years) asthma patients, who collected two or more drugs for obstructive airway diseases (ATC code R03) in any 1-year period during January 1st, 2017–2022 with at least 1 year coverage by a Belgian health insurance fund, we identified all patients eligible for biologics, based on the reimbursement criteria. Only patients who experienced at least one exacerbation (including OCS-treated and hospitalized exacerbations) in the year before their index date were eligible. Subjects with cystic fibrosis, lung cancer, or no dispensing of asthma maintenance therapy (treated only with bronchodilators) were excluded. Biologics prescribed by a dermatologist were not taken into account. Follow-up ended in case of event occurrence, death, emigration, or end of study period (January 1st, 2022), whichever came first.

### Exposure

Eligible patients who never used biologics were considered unexposed. Patients filling an anti-IgE (omalizumab–ATC R03DX05), or anti-IL5/IL5R prescription (benralizumab–ATC R03DX10, mepolizumab–ATC R03DX09 or reslizumab–ATC R03DX08) within the period of cohort entry, were considered exposed. Use of anti-IL4/IL-13 was not included, as this agent was not reimbursed for severe asthma in Belgium during the study period. The index date was defined as the dispensing date of the second outpatient delivery of a drug (ATC R03) leading to inclusion.

Additionally, incident biologic use was investigated, consisting of patients with a first dispensing of biologics and no dispensing of biologics in the year before study inclusion. For incident biologic users, the dispensing date of the first prescription of a biologic therapy during follow-up was considered as the index date. Patients with ≥2 prescription claims of different biologics at the treatment initiation date were excluded.

### Outcomes

Outcomes were identified using ICD-10 hospital discharge diagnoses and medical procedure codes, measured from the index date until end of follow-up. The incident date of outcomes was defined as the date of hospital admission in case of ICD codes, or the registration date for medical procedure codes. Outcomes were (acute) myocardial infarction, stroke (including ischemic and haemorrhagic stroke), arrythmia, congestive heart failure, peripheral artery disease, pulmonary embolism, and all-cause mortality. More information on outcome definitions is provided in eTable 2.

### Covariates

Baseline covariates were selected a priori based on their potential confounding effect. These covariates were age, sex, socio-economic status (SES), obesity or overweight, smoking status, dyslipidaemia, diabetes mellitus, upper respiratory comorbidities (allergic rhinitis, chronic rhinosinusitis, and/or nasal polyposis), allergic skin disease, upper gastro-intestinal tract disorder, hypertension, history of CV disease (including myocardial infarction, stroke, arrythmia, congestive heart failure, and peripheral artery disease), exacerbation history (one vs more exacerbations), medication history (including use of short-acting bronchodilators (SABD), maintenance therapy (beside biologics), use of antihistamines, and high OCS use), and frailty (based on the claims-based Frailty Indicator). The SES was based on the medical coverage and defined as low SES in case of increased reimbursements, which is in Belgium provided to individuals with low income. Comorbidities and medication history were identified in the year before follow-up using ICD-coded diagnoses, medical procedure codes, and/or ATC-coded medication dispensing claims. The IMA database represents all legal Belgium residents with Belgian health insurance and all hospitals in Belgium are obliged to make administrative, medical and nursing data available to the MHD centralized database. Therefore, this dataset is complete, and missing data was not applicable for the used covariates.

Patients were categorized as ever smoking if any ICD-code related to (history of) tobacco use or nicotine dependence, a nomenclature code for smoking cessation counseling or medication for smoking cessation was registered between January 1st, 2010, and the index date. Current smokers were ever smokers with active smoking ICD-coding before the index date and no smoking cessation attempts thereafter. Frailty was defined based on the John Hopkins claims-based Frailty Indicator (CFI)^21^^,^^22^ as it was not possible to define Fried's frailty phenotype using only administrative healthcare data. The CFI was developed to classify persons as frail and non-frail, validated against Fried's phenotype, and can be used in large datasets for confounding adjustment.^21^ This algorithm consists of 21 variables using only administrative data (including demographics, physical and cognitive dysfunction, and the Charlson Comorbidity Index) and uses a cut-off of ≥0.20 to define frail patients.^21^^,^^22^ More information on covariate definitions is provided in eTable 2.

### Statistical analysis

Mean and standard deviation, or median and interquartile range were presented for continuous variables, and counts and percentages for categorical variables. Crude event rates per outcome were computed as the total number of events per 100 person-years at risk. Inverse probability of treatment weighting (IPTW) was performed for each biologic therapy separately to minimize confounding by indication. Propensity scores (PS) were calculated using logistic regression models with the confounding covariates reported in Table 1 and above-described in the section covariates, stratified by calendar year (to account for over-time changes in prescribing behavior). Based on PS, stabilized weights were calculated to estimate the average treatment effect of the treated. Standardized mean differences (SMDs) for each covariate were computed to evaluate covariate balance before and after weighting, with a threshold of ≥0.1 to indicate imbalance between treatment groups, and graphically presented in Love plots. Weighted Cox proportional hazard regression models were used to compute adjusted hazard ratios (aHRs) with 95% confidence intervals (CIs) using robust variance estimators. Since all covariates were balanced, there were no additional variables incorporated in the Cox regression model. In detail, by using an intention-to-treat design, the relative risks of outcomes were assessed using cause-specific Cox regression models with death as a competing risk. The proportional hazard assumption was graphically checked. A two-sided p-value of <0.05 was considered statistically significant. All analyses were performed in R (version 4.3.0, Vienna, Austria).

**Table 1:** Baseline characteristics of the study population.

Patient characteristics	No biologics n = 167,641	Anti-IgE n = 1826	p-value^a^	Anti-IL5/IL5R n = 2398	p-value^a^
Age in years (SD)	63.9 (17.2)	53.7 (16.3)	<0.001	58.7 (14.5)	<0.001
Female	92,814 (55.4%)	1039 (56.9%)	0.197	1248 (52.0%)	0.001
Low SES	57,330 (34.2%)	485 (26.6%)	<0.001	644 (26.9%)	<0.001
Follow-up time in years (SD)	2.9 (1.5)	3.1 (1.5)		2.6 (1.4)	
Smoking status			<0.001		<0.001
No smoker	108,853 (64.9%)	1261 (69.1%)		1521 (63.4%)	
Past smoker	41,219 (24.6%)	415 (22.7%)		716 (29.9%)	
Current smoker	17,569 (10.5%)	150 (8.2%)		161 (6.7%)	
Obesity or overweight	17,050 (10.2%)	177 (9.7%)	0.527	299 (12.5%)	<0.001
Exacerbation history			<0.001		<0.001
One exacerbation	110,388 (65.8%)	819 (44.9%)		699 (29.1%)	
Two or more exacerbations	57,253 (34.2%)	1007 (55.1%)		1699 (70.9%)	
**Comorbidities**					
Upper respiratory	45,543 (27.2%)	771 (42.2%)	<0.001	1167 (48.7%)	<0.001
Allergic skin disease	30,683 (18.3%)	406 (22.2%)	<0.001	424 (17.7%)	0.450
Upper GTD	12,176 (7.3%)	142 (7.8%)	0.427	232 (9.7%)	<0.001
Dyslipidaemia	63,995 (38.2%)	481 (26.3%)	<0.001	804 (33.5%)	<0.001
Diabetes mellitus	32,890 (19.6%)	290 (15.9%)	<0.001	435 (18.1%)	0.074
Hypertension	62,053 (37.0%)	390 (21.4%)	<0.001	656 (27.4%)	<0.001
Prior CV disease	43,403 (25.9%)	241 (13.2%)	<0.001	438 (18.3%)	<0.001
Frailty	29,457 (17.6%)	54 (3.0%)	<0.001	102 (4.3%)	<0.001
**Drug use**					
Maintenance therapy (beside biologics)			<0.001		<0.001
ICS-LABA	103,776 (61.9%)	1115 (61.1%)		1504 (62.7%)	
ICS-LABA-LAMA	36,356 (21.7%)	488 (26.7%)		859 (35.8%)	
Other therapy	27,509 (16.4%)	223 (12.2%)		35 (1.5%)	
SABD use			<0.001		<0.001
Appropriate (0–2 canisters/year)	107,887 (64.4%)	839 (45.9%)		960 (40.0%)	
Overuse (3–5 canisters/year)	28,321 (16.9%)	326 (17.9%)		450 (18.8%)	
Heavy overuse (≥6 canisters/year)	31,433 (18.8%)	661 (36.2%)		988 (41.2%)	
≥6 packages OCS	10,298 (6.1%)	182 (10.0%)	<0.001	415 (17.3%)	<0.001
Antihistamines	60,989 (36.4%)	1229 (67.3%)	<0.001	1232 (51.4%)	<0.001

CV: cardiovascular, GTD: gastro-intestinal disorder, ICS: inhaled corticosteroids, IQR: interquartile range, LABA: long-acting beta2-agonists, LAMA: long-acting muscarinic antagonists, SABD: short-acting bronchodilators, SD: standard deviation.

Other therapy included ICS monotherapy, leukotriene receptor antagonist, cromoglicic acid or xanthine.

a p-value of the Chi-squared test for categorical variables, and of a t-test for continuous variables between the distributions of the no biologics group and the anti-IgE group, or the distributions of the no biologics group and the anti-IL5/IL5R group.

Sensitivity analyses were performed to check the robustness of results. To investigate new usership, analyses were repeated for incident biologic users only. To examine the impact of treatment response on the outcomes, analyses were repeated after stratification by treatment responder status. Patients were classified as responders if there was at least a 50% reduction in the exacerbation rate or a 50% reduction in OCS use (measured in defined daily dose (DDD)) in the first year of follow-up compared with the year before (baseline year). To reduce misclassification bias, analyses were repeated in patients without a disease label of chronic obstructive pulmonary disease (COPD), or by excluding patients above the age of 60 years. Since the COVID-19 pandemic overlapped with the study period, a sensitivity analysis was conducted by limiting the inclusion period to March 1st, 2020. Additionally, analyses were repeated using PS matching (PSM) with nearest neighbor matching without replacement, and a caliper of 0.05 with a 1:1 ratio. Finally, a nested case-control design was performed using multivariable conditional logistic regressions models, including the same covariates as the weighted Cox model. Cases (i.e., patients experiencing an outcome in the follow-up period) were randomly matched, using risk set sampling, with 3–4 controls based on age (±1 year), sex, and follow-up time. As an exploratory analysis of variables that may have improved after the initiation of anti-IgE or anti-IL5 therapy, we investigated significant interactions between treatment and hospitalized exacerbations (≥1 hospitalization), SABD overuse (≥3 canisters), frequent OCS use (≥6 filled prescriptions), and/or newly initiated CVD management (in those without prior CVD) for the risk of outcomes, by including these variables in the case-control analysis.

### Ethics approval

This study involving human participants used fully de-identified anonymized aggregated claims data for analyses and therefore ethics approval deemed unnecessary according to the national legalization (the Belgian Personal Data Protection Act (30th July 2018—2018/40581)) and the European legalization (GDPR (e.g., 2016/679—art. 5 and art. 89)). This study was approved by the IMA and MHD database administrators and by the Social Security and Health Chamber of the Belgian Information Security Committee (approval code IVC/KSZG/23/008), waiving the need for individual informed consents.^23^

### Role of the funding source

The study received no funding.

## Results

### Baseline characteristics

A total of 171,865 patients were included in the study (mean follow-up of 2.9 ± 1.5 years; 495,802 person-years), including 1826 patients treated with anti-IgE and 2398 patients with anti-IL5/IL5R, among whom 1559 (65.0%) treated with mepolizumab and 839 (35.0%) with benralizumab. Reslizumab treatment was not taken into account due to the low number of reslizumab users (<10) in our study population. Baseline characteristics are presented in Table 1. Before weighting, patients receiving biologic therapies were younger, less frail, experienced more frequent exacerbations, and had higher SABD use in the previous year, compared with the non-biologic group. Patients receiving anti-IgE therapy were more likely to be female, and had more allergic-driven characteristics, compared with non-biologic and anti-IL5/IL5R users. Patients in the anti-IL5/IL5R group, compared with the other patients, were more frequently classified as past smoker (while less current smoker), had higher OCS use, and received more frequently ICS-LABA-LAMA combination therapy. After weighting, covariates were well balanced. Love plots illustrating covariate balance before and after IPTW are shown in Supplemental Materials (eFigures 2 and 3).

### Anti-IgE therapy

**Fig. 1:**
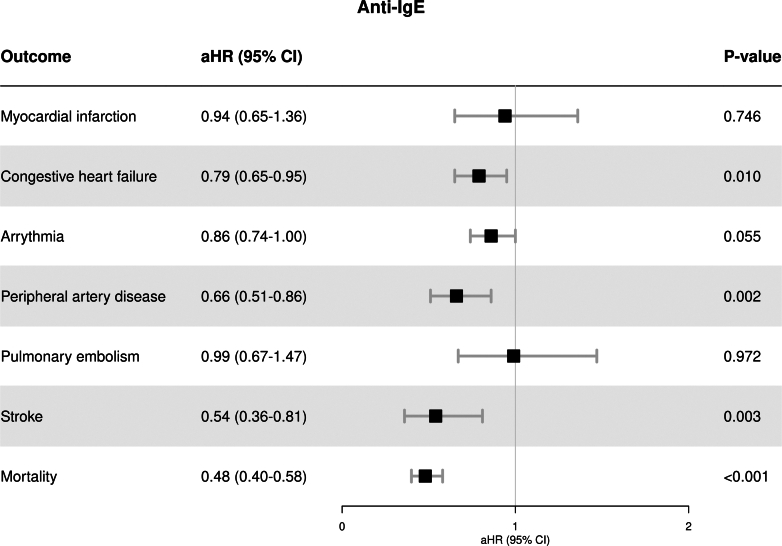
Cardiovascular risk and mortality in patients with asthma treated with anti-IgE versus non-biologic therapies after IPTW.

The number of events and crude event rates with 95% CI are presented in eTable 4. Overall, crude event rates were higher in the non-biologic group compared with the anti-IgE group. After multivariable adjustment using stabilized IPTW, anti-IgE therapy was associated with a significantly lower risk of congestive heart failure (aHR 0.79, 95% CI 0.65–0.95), peripheral artery disease (aHR 0.66, 95% CI 0.51–0.86), and stroke (aHR 0.54, 95% CI 0.36–0.81) compared with patients without biologic therapy. Moreover, anti-IgE therapy was associated with a significantly reduced mortality risk (aHR 0.48, 95% CI 0.40–0.58) compared with patients without biologic therapy. No significant differences in the risk of myocardial infarction, arrythmia, and pulmonary embolism were observed between anti-IgE compared with non-biologic users. Fig. 1 shows the aHRs after IPTW for each outcome evaluated.

### Anti-IL5/IL5R therapy

**Fig. 2:**
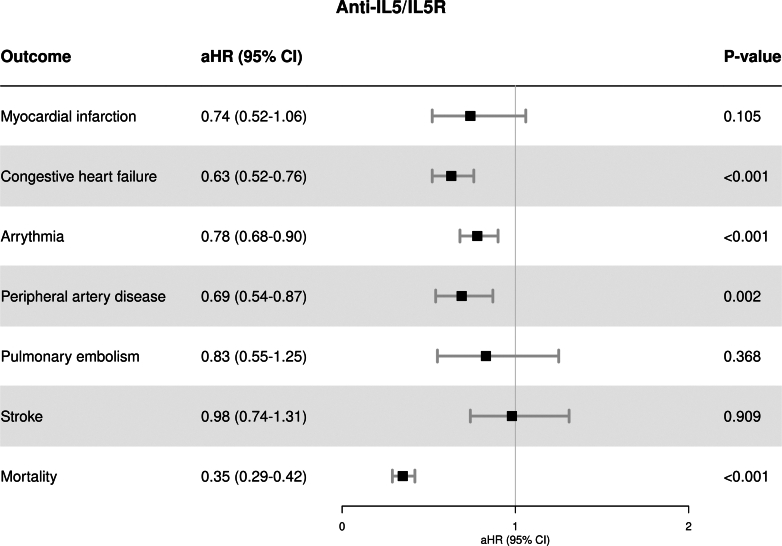
Cardiovascular risk and mortality in patients with asthma treated with anti-IL5/IL5R versus non-biologic therapies after IPTW.

Crude event rates were higher in the non-biologic group compared with the anti-IL5/IL5R group (eTable 4). After weighting, anti-IL5/IL5R therapy was associated with a significantly lower risk of congestive heart failure (aHR 0.63, 95% CI 0.52–0.76), arrythmia (aHR 0.78, 95% CI 0.68–0.90), and peripheral artery disease (aHR 0.69, 95% CI 0.54–0.87) compared with non-biologic users. Moreover, anti-IL5/IL5R was associated with a 65% lower risk of death (aHR 0.35, 95% CI 0.29–0.42) compared with patients not receiving biologics. No statistically significant differences were observed for the association with myocardial infarction, pulmonary embolism, and stroke. Fig. 2 shows the aHRs for each outcome evaluated.

### Sensitivity analysis

Results were in line with those from additional analyses in patients with incident biologic use, when excluding patients with COPD (by disease label or excluding those above the age of 60 years), when restricting the inclusion period to March 1st, 2020, when using the 1:1 nearest neighbor PSM approach, and the nested case-control design (eTables 5–9). Although CV disease event rates were four times lower (power loss) in patients aged ≤60 years, potential protective effects were maintained (especially on mortality, and additionally for anti-IL5/IL5R use on pulmonary embolism). The analysis stratified by treatment response shows that potential protective effects of the investigated biologic therapies on mortality were not limited to patients halving their exacerbations or OCS use (eTable 10). In the case-control analysis, we observed that anti-IL5 use significantly reduced heart failure by reducing SABD overuse (eTable 11). In contrast, patients who despite their therapy still experienced hospitalized exacerbations, had a higher risk of stroke associated with anti-IgE therapy and a higher risk of PAD associated with anti-IgE or anti-IL5 therapy.

## Discussion

In this nationwide cohort study including approximately 1800 users of anti-IgE and around 2400 anti-IL5/IL5R users, biologic therapies were associated with a significantly lower risk of CV events and mortality compared with non-biologic users. Anti-IgE was associated with a lower risk of congestive heart failure, peripheral artery disease, and stroke, whereas anti-IL5/IL5R was associated with a lower risk of congestive heart failure, arrythmia, and peripheral artery disease.

Research has demonstrated that uncontrolled or severe asthma is associated with an increased risk of CV disease.^6^, ^7^, ^8^, ^9^ Underlying mechanisms are still not fully understood, but proposed mechanisms include chronic inflammation, impaired lung function, use of asthma medications (mainly OCS), and shared risk factors.^7^, ^8^, ^9^^,^^24^ Consequently, obtaining good asthma control might potentially reduce CV risk in severe asthma patients. The CV risk following an exacerbation has been studied mainly in COPD patients, showing mostly an elevated risk of CV events following a COPD exacerbation.^25^^,^^26^ Moreover, both short-term and long-term use of OCS have been associated with an increased risk for developing CV events.^27^^,^^28^ Both anti-IgE and anti-IL5/IL5R treatment demonstrated significant reductions in exacerbations in patients with severe asthma,^10^ possibly preventing elevated CV risks after exacerbations and avoiding the need for OCS bursts. However, our additional analyses suggest that cardiovascular protective effects may be achieved by e.g., controlling chronic inflammatory pathways rather than solely reducing acute exacerbation events or OCS use.

Indeed, it can be suggested that by reducing inflammatory markers, the risk of developing CV disease may be diminished. IgE activates mast cells and induces the production of pro-inflammatory cytokines, including IL-6.^7^ Elevated levels of inflammatory biomarkers, such as C-reactive protein (CRP) and IL-6, are reported in patients with severe asthma.^29^ IL-6 has been shown to be positively linked with atherosclerosis and RCTs of treatments targeting the IL-6 pathway (e.g., ziltivekimab) in patients with high risk of atherosclerotic diseases demonstrated a reduction in CV event rates.^30^ However, to the best of authors' knowledge there are no studies demonstrating a clinical relevant reduction of IL-6 serum levels after anti-IgE treatment in patients with asthma.

EXCELS, a post-marketing safety study of omalizumab (anti-IgE therapy), reported higher arterial thromboembolic events compared with non-users, whereas we found no significant association between omalizumab and myocardial infarction (possibly due to lower event rates, reducing statistical power), and a lower risk of stroke.^13^ The elevated risk in the EXCELS study was attributed to higher blood IgE levels and more patients with severe asthma at baseline for the omalizumab group.^6^^,^^13^ After adjusting the analysis for asthma severity, the results were no longer statistically significant.^13^ Moreover, a pooled analysis of 25 RCTS and two omalizumab extension studies with a mean duration of 0.6 years found no evidence for an elevated CV risk.^17^ Additionally, in open-label extension studies for benralizumab and mepolizumab serious CV events such as myocardial infarction, deep venous thrombosis and stroke have been reported.^20^^,^^31^^,^^32^ In our case-control analysis with interaction terms, we observed that anti-IgE was associated with a higher risk of myocardial infarction, and both anti-IgE and anti-IL5 were associated with a higher risk of pulmonary embolism, and anti-IL5 with stroke, however, none of these associations were statistically significant.

Another potential mechanism is the reduction of eosinophils, which are almost completely depleted with benralizumab and significantly reduced with mepolizumab.^33^ The association of eosinophils and CV disease is still unexplained, some studies observed a protective effect of eosinophils on myocardial infarction and stroke, while other studies found an increased risk of CV disease with higher blood eosinophil counts.^34^ Xu et al. postulate that eosinophils might be protective in acute events but that eosinophils contribute to calcification and atherosclerosis in chronic CV settings.^34^ In our study, we only found a statistically significant reduction in chronic CV diseases, such as arrythmia, heart failure, and peripheral artery disease for all of the investigated biologics compared with non-biologic users. More research is needed to increase our understanding of underlying pathophysiological and (anti)inflammatory mechanisms.

Strengths of this nationwide cohort study include the large sample size, the use of nationally representative data, information on ambulatory as well as hospital data, and statistical adjustment to minimize confounding by indication using stabilized IPTW. Nevertheless, our study had also limitations. As in all observational studies, the observed associations—between use of biologic therapies and reduced cardiovascular events and mortality in patients with severe asthma–could not be proven causal, and residual confounding may still be present. Replication in other databases should confirm the external validity. Nevertheless, we weighted our analyses for differences in 18 variables and additional sensitivity analyses did not suggest that biologic users were simply a population at lower risk. Second, coding errors and misclassification bias may be present due to the observational nature of our study, as well as preferential prescribing of biologics. Third, although we controlled for multiple confounders, the probability of unmeasured confounding due to missing lifestyle characteristics (e.g., body weight, smoking pack-years) and clinical values (e.g., blood values, spirometry) cannot be fully ruled out. Fourth, we were not able to assess the exact asthma endotype as we lacked information about blood eosinophil counts and FeNO levels, although we tried to mitigate this limitation by including allergic-driven comorbidities and proxies for asthma severity (such as OCS use, SABD overuse, and exacerbation history). Also in this regard, we did not perform head-to-head comparisons between anti-IgE and anti-IL5/IL5R therapy. Therefore, these results cannot be used to directly compare anti-IgE treatment with anti-IL5/IL5R treatment. Moreover, since medication use was based on dispensing data, we were not able to confirm if the patient actually used the dispensed medication. Furthermore, smoking status was based on smoking cessation attempts and hospital-registered codes, potentially underestimating the amount of smokers. We identified frailty based on the validated John Hopkins claims-based Frailty Indicator using only administrative data.^21^ This measure may have a high specificity but a lower sensitivity, potentially underestimating the amount of frail patients. Lastly, all-cause mortality was investigated as we were not able to study cardiovascular-related mortality, which is often included in the investigation of major adverse cardiovascular events (MACE), because information on reason of death was lacking.

In conclusion, biologic therapies in adults with severe asthma were associated with a significantly lower risk of cardiovascular events and mortality compared with non-biologic users. Anti-IgE was associated with a lower risk of congestive heart failure, peripheral artery disease, and stroke, whereas anti-IL5/IL5R therapy was associated with a lower risk of congestive heart failure, arrythmia, and peripheral artery disease compared with similar eligible non-biologic users. Further research, and external validation, is needed to elucidate whether this intriguing association is truly causal.

## Contributors

FVV and LL had direct access to the data, verified underlying reported data, and contributed to the concept and design of the study. FVV performed the statistical analysis, interpretation, and writing under supervision of LL. DV, MG, LV, GB, and LL provided feedback to optimize the design of the study. DV, MG, LV, GB, and LL revised the manuscript critically. All authors contributed to the article and approved the final version of the manuscript.

## Data sharing statement

The data that support the findings of this study are available from the InterMutualistic Agency and Minimal Hospital Dataset, but restrictions apply to the availability of these data, which were used under license for the current study, and so are not publicly available. Any further inquiries regarding data availability should be directed to Professor Lies Lahousse (lies.lahousse@ugent.be) or the administrators of the InterMutualistic Agency (IMA) database or Minimal Hospital Dataset.

## Declaration of interests

Outside this manuscript, LL has been consulted as expert for AstraZeneca, GlaxoSmithKline and Sanofi, and has given lectures sponsored by Chiesi, Johnson and Johnson, IPSA vzw and Domus Medica vzw (non-profit organizations facilitating lifelong learning for health care providers), all paid to her institution. She received support for travel from Menarini. None of which are related to the content of this work. Outside this manuscript, LV received research grants from The Family Kamprad Foundation, Svensk Lungmedicinsk Förening, the Swedish government and country council ALF grant, The Swedish Heart and Lung Foundation and AstraZeneca, all paid to his institution. LV received payments or honoraria for lectures or presentations by GSK, Astrazeneca, Boehringer, Novartis, Chiesi, Resmed, Pulmonx, Grifols, and Sanofi. GB received fees for advisory boards and lectures from AstraZeneca, Chiesi, GlaxoSmithKline, and Sanofi Regeneron. All other authors declare that the research was conducted in the absence of any commercial or financial relationships that could be construed as a potential conflict of interest.

## Appendix A Supplementary data

Supplementary eTables and eFigures
