# Supplementary material for: Cardiovascular safety of biologic therapies in patients with severe asthma: a nationwide cohort study in Belgium
Source: Lancet Reg Health Eur. 2025 Aug 6;57:101420. doi: 10.1016/j.lanepe.2025.101420 (PMC12355080; doi:10.1016/j.lanepe.2025.101420)
Supplement: Supplementary eTables and eFigures [file mmc1.pdf]

Supplemental materials

Cardiovascular safety of biologics in patients with severe asthma: a nationwide cohort study

Table of contents

Supplemental tables 3
eTable 1: Strengthening the Reporting of Observational Studies in Epidemiology (STROBE) guideline 3
eTable 2: Definition of in- and exclusion criteria, comorbidities, comedication and clinical risk scores 5
eTable 3: Baseline characteristics of the study population with SMD before and after weighting. 12
eTable 4: Crude event rates per outcome stratified by treatment. 14
eTable 5: Sensitivity analysis – aHR of outcomes after IPTW in patients with incident biologic use. 14
eTable 6: Sensitivity analysis – aHR of outcomes after IPTW in patients without COPD disease label or aged ≤60 years. 15
eTable 7 Sensitivity analysis – aHR of outcomes after IPTW in patients with inclusion between January, 2017 and March, 2020. 15
eTable 8: Sensitivity analysis – HR of outcomes after PSM. 15
eTable 9: Sensitivity analysis – aOR of outcomes (nested case-control analysis) 16
eTable 10: Sensitivity analysis – aHR of outcomes after IPTW, stratified by treatment response 16
eTable 11: Sensitivity analysis – aOR of outcomes (nested case-control analysis with interaction terms) 17
eTable 12: Coefficients from the PS model. 18
Supplemental figures 19
eFigure 1: Flowchart of inclusion of study population 19
eFigure 2: Love plot of propensity score model for anti-IgE use 20
eFigure 3: Love plot of propensity score model for anti-IL5/IL5R use 21
eFigure 4: Mirrored histogram plot of propensity scores of anti-IgE therapy after adjustment. 22
eFigure 5: Mirrored histogram plot of propensity scores of anti-IL5/IL5R therapy after adjustment. 22
References 23

Supplemental tables

eTable 1: Strengthening the Reporting of Observational Studies in Epidemiology (STROBE) guideline

eTable 1: Compliance to the STROBE (Strengthening the Reporting of Observational Studies in Epidemiology) reporting guideline [1].

	Item No.	Recommendation	Page No.
**Title and abstract**	1	(*a*) Indicate the study's design with a commonly used term in the title or the abstract	1
		(*b*) Provide in the abstract an informative and balanced summary of what was done and what was found	2
Background/rationale	2	Explain the scientific background and rationale for the investigation being reported	5
Objectives	3	State specific objectives, including any prespecified hypotheses	5-6
Study design	4	Present key elements of study design early in the paper	6-7
Setting	5	Describe the setting, locations, and relevant dates, including periods of recruitment, exposure, follow-up, and data collection	6-7
Participants	6	(*a*) Give the eligibility criteria, and the sources and methods of selection of participants. Describe methods of follow-up	6-7
Variables	7	Clearly define all outcomes, exposures, predictors, potential confounders, and effect modifiers. Give diagnostic criteria, if applicable	8, eTable 2
Data sources/ measurement	8*	For each variable of interest, give sources of data and details of methods of assessment (measurement). Describe comparability of assessment methods if there is more than one group	7-9, eTable 2
Bias	9	Describe any efforts to address potential sources of bias	9-10
Study size	10	Explain how the study size was arrived at	eFigure 1
Quantitative variables	11	Explain how quantitative variables were handled in the analyses. If applicable, describe which groupings were chosen and why	9-10
Statistical methods	12	(*a*) Describe all statistical methods, including those used to control for confounding	9-10
		(*b*) Describe any methods used to examine subgroups and interactions	10
		(*c*) Explain how missing data were addressed	8
		(*d*) If applicable, explain how loss to follow-up was addressed	n.a.
		(*e*) Describe any sensitivity analyses	10
Participants	13*	(a) Report numbers of individuals at each stage of study—eg numbers potentially eligible, examined for eligibility, confirmed eligible, included in the study, completing follow-up, and analysed	11, eFigure 1
		(b) Give reasons for non-participation at each stage	/
		(c) Consider use of a flow diagram	eFigure 1

Descriptive data	14*	(a) Give characteristics of study participants (eg demographic, clinical, social) and information on exposures and potential confounders	Table 1, eFigure 2, eFigure 3
		(b) Indicate number of participants with missing data for each variable of interest	/
		(c) Summarise follow-up time (eg, average and total amount)	11, Table 1
Outcome data	15*	Report numbers of outcome events or summary measures over time	eTable 3
Main results	16	(*a*) Give unadjusted estimates and, if applicable, confounder-adjusted estimates and their precision (eg, 95% confidence interval). Make clear which confounders were adjusted for and why they were included	12, Figure 1, Figure 2
		(*b*) Report category boundaries when continuous variables were categorized	/
		(*c*) If relevant, consider translating estimates of relative risk into absolute risk for a meaningful time period	/
Other analyses	17	Report other analyses done—eg analyses of subgroups and interactions, and sensitivity analyses	12-13, eTable 5-11
Key results	18	Summarise key results with reference to study objectives	13
Limitations	19	Discuss limitations of the study, taking into account sources of potential bias or imprecision. Discuss both direction and magnitude of any potential bias	13-16
Interpretation	20	Give a cautious overall interpretation of results considering objectives, limitations, multiplicity of analyses, results from similar studies, and other relevant evidence	16
Generalisability	21	Discuss the generalisability (external validity) of the study results	15-16
Funding	22	Give the source of funding and the role of the funders for the present study and, if applicable, for the original study on which the present article is based	11

eTable 2: Definition of in- and exclusion criteria, comorbidities, comedication and clinical risk scores

VARIABLE	ICD, ATC AND MEDICAL PROCEDURE CODES
**INCLUSION CRITERIA** [2,3]	
Asthma maintenance therapy	**ATC:**<br>*Inhaled corticosteroids ~ ATC: R03BA01, R03BA02, R03BA05*<br>*Combination therapy of long-acting β2-agonists (ATC: R03AC12, R03AC13, R03AC18, R03AC19) and inhaled corticosteroids ~ due to use of the ATC for the specific categories or use of ATC: R03AK06 – R03AK08, R03AK10 – R03AK12, R03AK14*<br>*Anti-IgE: R03DX05*<br>*Anti-IL5/IL5R: R03DX08-R03DX10*<br>*Multiple R03 medication: R03AL08, R03AL09, R03AL11, R03AL12, R03BC01, R03CC02, R03CC03, R03DA01, R03DA04, R03DC01, R03DC03, R03DC53*
≥18 years	Age ≥18 years on index date
≥1 year coverage	≥1 year coverage by a Belgian health insurance fund
Exacerbation history	One of the following events:<br>• An outpatient prescription fill for an oral corticosteroid (OCS) ~ **ATC**: H02AB04, except parenteral use (defined on package level). Prescription fills needed to be separated by at least 14 days to be considered as separate events. OCS prescription fill followed by a hospital admission or emergency department (ED) visit for a severe exacerbation within 14 days were counted only as one exacerbation, to avoid counting exacerbations twice.<br>• A hospital admission or ED visit with an asthma or (acute) COPD exacerbation (ICD-10: J44.0, J44.1, J45.X1, J45.X2, J45.901 or J45.902) or with a primary diagnosis code for chronic lower respiratory disease (ICD-10: J40 – J47) or respiratory failure (ICD-10: J96) and a secondary diagnosis code for asthma or (acute) COPD exacerbation. Hospitalizations or ED visits with concomitant pneumonia diagnosis were excluded. **ICD-10**: A01.03, A02.22, A37.01, A37.11, A37.81, A37.91, A50.04, A54.84, B01.2, B05.2, B06.81, B77.81, J09.X1, J09.X2, J09.X3, J10.0, J11.0, J12, J13, J14, J15, J16, J17, J18, J84.11, J84.2, J85.1, J95.851.<br><br>Categorized as one exacerbation, or two or more exacerbations in the year before follow-up.
**EXCLUSION CRITERIA**	
Cystic fibrosis	**ICD-10:** E84<br>**ATC**: R05CB13, R07AX02, R07AX30, R07AX31, R07AX32<br>**Medical procedure code:** 604450, 604472, 604494, 604516, 604531, 604553
Lung cancer	**ICD-10:** C34, C38.4, C78.0, C78.2<br>**Medical procedure code:** 227216, 227220, 594311, 594322, 594333, 594344, 594355 ,594366, 594370, 594381
Treatment with only bronchodilators	**ATC:** *Short-acting bronchodilators ~ ATC: R03AC02, R03AC03, R03AL01, R03AL02, R03BB01*<br>*Long-acting bronchodilators ~ ATC: R03AC12, R03AC13, R03AC18, R03AC19, R03AL03-R03AL06, R03BB04-R03BB07*
**DEMOGRAFICS (on index date)**	
Age	Age on index date based on the year and month of birth, not the exact date due to patient privacy.
Sex	Sex on index date
Socio-economic status	Binomial variable derived from medical coverage, based on copayments for medical procedures and medication at the index date.

**SMOKING STATUS (from January 1st, 2010 till follow-up) [2-4]**	
Smoking status	**ICD-9:** 305.1, V15.82<br>**ICD-10:** F17, Z71.6, Z72.0, Z87.891<br>**ATC**:N06AX12, N07BA<br>**Medical procedure code:** 740434, 740445, 740456, 740460, 740471, 740482<br><br>Categorized as past smoker if:<br>• Past smoking ICD before index date<br>o Past smoking ICD: history of tobacco use ~ **ICD-9**: V15.82 or **ICD-10**: F17.201, F17.211, F17.221, F17.291, Z87.891<br>o Current smoking ICD: **ICD-9**: 305.1 or **ICD-10**: F17.200, F17.203, F17.208, F17.209, F17.210, F17.213, F17.218, F17.219, F17.220, F17.228, F17.290, F17.293, F17.298, F17.299, Z71.6, Z72.0<br>• Last smoking cessation attempt before index date and no current smoking ICD thereafter: **ATC**: N06AX12, N07BA or **Medical procedure code**: 740434, 740445, 740456, 740460, 740471, 740482.
**PATIENT CHARACTERISTICS (≤1 year before follow-up)**	
Obesity or overweight	**ICD-10:** E66, Z68.25-Z68.29, Z68.3, Z68.4<br>**ATC:** A08AA62, A08AB01<br>**Medical procedure code:** 241776, 241780, 241813, 241824, 241835, 241846
**COMORBIDITIES (≤1 year before follow-up) [2-4]**	
Dyslipidaemia	**ICD-10:** E78<br>**ATC:** C10
Upper respiratory (allergic rhinitis, chronic rhinosinusitis or nasal polyposis)	**ICD-10:** J30, J32, J33<br>**ATC:** R01AD
Allergic skin disease	**ICD-10:** L20-L30, L50<br>**ATC:** D07A
Upper gastro-intestinal tract disorder	**ICD-10:** K20, K21, K22.1, K22.7, K25-K29, Z87.11<br>**ATC:** A02BD04, A02BD08, A02BD11<br>**Medical procedure code:** 172616, 172620, 172631, 172642, 172653, 172664, 474854, 474865, 550093, 550104, 552370, 552381
Arrythmia	**ICD-10:** I44, I45, I46, I47, I48, I49, R00.0, R00.1, R00.8, T82.1, Z45.0, Z95.0<br>**ATC:** C01EB10, C01B, C07AA07, C07BA07, C07FX02<br>**Medical procedure code:** 180272, 180283, 180294, 180305, 589514, 589525, 589551, 589562
Myocardial infarction	**ICD-10:** I21, I22, I25.2
Congestive heart failure	**ICD-10:** I09.81, I11.0, I13.0, I13.2, I25.5, I42.0, I42.6-I42.9, I43, I50, P29.0<br>**ATC: combination of treatment of all of the following drug classes [5]:**<br>**I) Beta blocker (selective or alpha and beta blocking):** C07AB, C07AG, C07BB, C07BG, C07CB, C07CG, C07DB, C07FB, C07FX03, C07FX04, C07FX05, C07FX06<br>**II) ACE inhibitor or angiotensin II receptor blocker:** C09A, C09BA, C09BB, C09C, C09DA, C09DB, C09DX01, C09DX02, C09DX03, C09DX04, C09DX06, C09DX07, C09DX08, C10BX04, C10BX06, C10BX07, C10BX10, C10BX11, C10BX12, C10BX13, C10BX14, C10BX15, C10BX16, C10BX17, C10BX18, C10BX19, C10BX20, C10BX21<br>**III) Loop diuretic:** C03C, C03EB<br>**or use of ≥1 combination product (beta blocker + ACE inhibitor or beta blocker + angiotensin II receptor blocker)**: C09BX02, C09BX04, C09BX05, C09BX06, C09BX07, C09DX05 **and loop diuretic**
Peripheral vascular disease	**ICD-10:** I70, I71, I73.1, I73.8, I73.9, I74, I77, I79.0, K55.1, K55.8, K55.9, Z95.82, Z98.62<br>**Medical procedure code:** 229294, 229305, 229316, 229320, 229331, 229342, 235071, 235082, 235093, 235104, 235115, 235126, 235196, 235200, 235211, 235222, 236014, 236025, 236036, 236040, 236051, 236062, 237016, 237020, 237031, 237042, 237053, 237064, 237075, 237086, 237090, 237101, 237171, 237182, 589050, 589061, 589094, 589105, 589175, 589186, 589595, 589606, 589610, 589621, 589632, 589643, 589654, 589665
Cerebrovascular disease	**ICD-10:** G45, G46, H34.0, I60, I61, I62, I63, I65, I66, I67, I68, I69, Z86.73<br>**Medical procedure code:** 182136, 182140, 182151, 182162, 182173, 182184, 477724, 477746, 477761, 477783

Stroke	**ICD-10:** I61, I62, I63, I67.89, I69.1, I69.2, I69.3, I69.8, I69.9, I97.82, Z86.73<br>**Medical procedure code:** 182136, 182140, 182151, 182162, 182173, 182184, 477724, 477746, 477761, 477783
Hypertension	**ICD-10:** I10, I11, I12, I13, I15, I16, I67.4<br>**ATC: combination of treatment of ≥1 of the following drug classes**:<br>**I) Cardioselective beta blocker:** C07AB, C07FX03, C07FX04, C07FX05, C07FX06<br>**II) ACE inhibitor or angiotensin II receptor blocker:** C09A, C09B, C09C, C09D, C10BX04, C10BX06, C10BX10, C10BX12, C10BX15, C10BX16, C10BX17, C10BX20, C10BX21<br>**III) Calcium channel blocker:** C08C, C08G, C09XA53, C10BX03, C10BX09<br>**IV) Non-loop diuretic:** C03A, C03BA, C03BB, C03EA, C07BA, C07BG, C07DA, C09XA52<br>**V) Other antihypertensive drugs (alpha adrenergic blocker, vasodilator):** C02A, C02B, C02C, C02DB, C02DD, C02DG, C02L<br>**Combination product:** C07B, C07CB, C07DB, C07FB, C09XA54, C10BX07, C10BX11, C10BX13, C10BX14, C10BX18, C10BX19
Pulmonary heart disease	**ICD-10:** I26, I27, I28, Z86.711<br>**ATC:** C02KX, B01AC11, B01AC21, B01AC27<br>**Medical procedure code:** 211540, 211562
Dementia	**ICD-10:** A81.0, F01, F02, F03, F05, G30, G31.0, G31.83, G31.85<br>**ATC**: N06D
Connective tissue disease	**ICD-10:** M05.0, M05.1, M05.2, M05.3, M05.8, M05.9, M06.0, M06.3, M06.9, M32, M33, M34, M35, M36.0, M36.8
Peptic ulcer disease	**ICD-10**: K22.1, K25, K26, K27, K28, Z87.11<br>**ATC:** A02BD04, A02BD08, A02BD11<br>**Medical procedure code:** 550093, 550104, 552370, 552381
Mild liver disease	**ICD-10:** B17.0, B17.10, B18, B19.10, B19.20, K70.0, K70.1, K70.2, K70.3, K70.9, K71.3, K71.4, K71.5, K71.6, K71.7, K71.8, K71.9, K73, K74, K75.2, K75.3, K75.4, K75.8, K75.9, K76.0, K76.2, K76.3, K76.4, K76.89, K76.9, Z94.4<br>**ATC:** J05AF07, J05AF08, J05AF10, J05AP<br>**Medical procedure code:** 318076, 318080, 318334, 318345, 472113, 472124, 556754, 556765, 556776, 556780, 589352, 589363
Diabetes mellitus (DM)	**ICD-10:** E08, E09, E10, E11, E13, E14, Z46.81, Z96.41<br>**ATC**: A10<br>**Medical procedure code:** 102852, 107015, 107030, 107052, 107074, 109594, 174370, 174381, 174392, 174403, 174414, 174425, 174436, 174440, 174451, 174462, 174473, 174484, 174495, 174506, 174510, 174521, 423135, 423150, 423172, 423194, 423216, 423231, 423334, 423813, 423835, 423850, 433554, 433565, 540772, 540783, 543712, 543723, 653671, 653682, 697093, 697104, 754176, 754191, 754250, 754272, 754736, 757352, 757374, 757396, 757411, 757514, 757536, 757551, 757573, 770033, 770055, 770070, 771573, 771595, 773393, 773496, 784630, 784641, 784652, 784663, 785735, 785750, 785772, 785794, 785816, 785831, 785853, 785875, 785890, 785912, 785934, 785956, 786015, 786030, 786100, 788756, 788771, 788793, 788815, 788830, 788852, 788874, 788896, 788911, 788933, 788955, 789751, 789773, 789795, 789810, 789832, 789854, 789876, 789891, 789913, 789935, 794032, 794054, 794076, 794091, 794253, 794275, 794290, 794312, 794334, 794356, 794371, 794393, 794415, 794430, 794452, 961295, 961306, 961332, 961343
Diabetes mellitus with vascular complications	**ICD-10:** E08.2, E08.3, E08.4, E08.5, E08.8, E09.2, E09.3, E09.4, E09.5, E09.8, E10.2, E10.3, E10.4, E10.5, E10.8, E11.2, E11.3, E11.4, E11.5, E11.8, E13.2, E13.3, E13.4, E13.5, E13.8<br>**Medical procedure code:** 653671, 653682, 697093, 697104, 770070, 773393, 773496
Hemiplegia/paraplegia	**ICD-10:** G04.1, G11.4, G80.0, G80.1, G80.2, G81, G82, G83.0, G83.4, G83.9<br>**Medical procedure code:** 643414, 643425
Chronic kidney disease	**ICD-10:** I12.0, I13.11, I13.2, N03.2, N03.3, N03.4, N03.5, N03.6, N03.7, N05.2, N05.3, N05.4, N05.5, N05.6, N05.7, N18, N19, N25.0, T86.1, Z48.22, Z49, Z91.15, Z94.0, Z99.2<br>**Medical procedure code:** 107096, 107111, 107133, 107155, 318010, 318021, 318290, 318301, 470293, 470304, 470315, 470326, 470330, 470341, 470352, 470374, 470385, 470400, 470422, 470433, 470444, 470466, 470470, 470481, 470875, 470890, 470901, 470912, 470934, 470945, 471111, 471122, 471133, 471144, 471155, 471166, 471170, 471181, 474714, 474725, 754294, 757433, 757492, 761272, 761283, 761456, 761471, 761493, 761515, 761526, 761530, 761552, 761574, 761596, 761655, 761670, 767594, 767616, 767631, 767664, 767686, 767701, 767723, 767734, 767756, 767782, 767804, 767815, 767826, 767830, 767841, 767955, 767966

Cancer	**ICD-10:** C00-C96, Z51.0, Z51.11, Z51.12<br>**ATC:** L01<br>**Medical procedure code:** 154873, 154884, 154895, 154906, 157231, 157242, 201191, 201202, 201213, 201224, 220275, 220286, 220371, 220382, 226914, 226925, 226936, 226940, 227216, 227220, 227275, 227286, 227636, 227640, 227651, 227662, 227673, 227684, 227695, 227706, 227710, 227721, 227732, 227743, 227754, 227765, 227776, 227780, 227791, 227802, 227813, 227824, 227835, 227846, 230473, 230484, 231033, 231044, 235152, 235163, 241231, 241242, 241415, 241426, 241430, 241441, 241452, 241463, 242012, 242023, 242034, 242045, 242292, 242303, 242314, 242325, 242830, 242841, 242852, 242863, 242874, 242885, 242896, 242900, 243051, 243062, 243073, 243084, 243235, 243246, 243736, 243740, 243751, 243762, 243773, 243784, 244016, 244020, 244031, 244042, 244075, 244086, 244311, 244322, 244856, 244860, 244893, 244904, 244915, 244926, 244930, 244941, 244952, 244963, 244974, 244985, 245512, 245523, 245534, 245545, 246050, 246061, 246072, 246083, 247111, 247122, 247133, 247144, 251753, 251764, 251775, 251786, 254892, 254903, 256115, 256126, 256336, 256340, 256572, 256583, 256771, 256782, 257191, 257202, 258355, 258366, 258370, 258381, 258392, 258403, 258451, 258462, 258554, 258565, 258856, 258860, 258871, 258882, 258893, 258904, 259033, 259044, 259114, 259125, 260190, 260201, 260411, 260422, 260433, 260444, 260551, 260562, 260654, 260665, 260750, 260761, 261111, 261122, 261391, 261402, 261472, 261483, 261671, 261682, 261774, 261785, 261796, 261800, 262334, 262345, 262570, 262581, 277756, 277760, 277771, 277782, 278795, 278806, 278810, 278821, 280136, 280140, 280151, 280162, 281831, 281842, 281956, 281960, 282310, 282321, 282671, 282682, 288455, 288466, 288470, 288481, 310494, 310505, 311312, 311323, 312550, 312561, 312572, 312583, 312594, 312605, 312653, 312664, 312970, 312981, 317111, 317122, 350070, 350092, 350114, 350125, 350136, 350140, 350232, 350254, 350265, 350276, 350280, 350291, 350302, 350372, 350383, 350394, 350405, 350416, 350420, 350674, 350685, 350696, 350700, 431174, 431185, 433016, 433020, 435831, 435842, 436295, 436306, 436376, 436380, 444113, 444124, 444135, 444146, 444150, 444161, 444172, 444183, 444194, 444205, 444216, 444220, 444231, 444242, 444253, 444264, 444275, 444286, 444290, 444301, 444312, 444323, 444334, 444345, 444356, 444360, 444371, 444382, 444393, 444404, 444415, 444426, 444430, 444441, 444452, 444463, 444474, 444485, 444496, 444500, 444511, 444522, 444533, 444544, 444555, 444566, 444570, 444581, 444592, 444603, 444636, 444640, 444651, 444662, 444673, 444684, 473970, 473981, 474795, 474806, 532696, 532700, 532711, 532722, 548575, 548586, 565073, 565084, 565095, 565106, 565110, 565121, 565132, 565143, 565154, 565165, 587834, 587845, 587871, 587882, 587893, 587904, 587915, 587926, 588431, 588442, 588453, 588464, 588475, 588486, 588490, 588501, 588512, 588523, 588534, 588545, 588556, 588560, 588571, 588582, 588593, 588604, 588770, 588781, 588976, 588980, 589691, 589702, 589713, 589724, 589831, 589842, 589875, 589886, 594252, 594263, 594274, 594285, 594296, 594300, 594311, 594322, 594333, 594344, 594355, 594366, 594370, 594381, 594392, 594403, 594414, 594425, 594436, 594440, 594451, 594462, 594495, 594506, 594510, 594521, 594532, 594543, 594554, 594565, 594576, 594580, 594591, 594602, 594613, 594624, 594635, 594646, 594694, 594705, 594716, 594720, 594753, 594764, 594775, 594786, 594790, 594801, 594812, 594823, 594834, 594845, 594856, 594860, 594871, 594882, 594893, 594904, 594915, 594926, 594930, 594941, 597273, 597295, 598581, 745010, 745021, 745032, 745043, 745113, 745124, 745135, 745146, 745150, 745161, 771632, 771643
Any malignancy including leukaemia and lymphoma	**ICD-10:** C00-C76, C80.1, C80.2, C81-C96, Z51.0, Z51.11, Z51.12<br>**ATC:** L01<br>**Medical procedure code:** see 'Cancer'
Metastatic cancer	**ICD-10:** C77-C79, C80.0
Moderate or severe liver disease	**ICD-10:** B15.0, B16.0, B16.2, B17.11, B19.0, B19.11, B19.21, I85, I86.4, K70.4, K71.1, K72.1, K72.9, K76.5, K76.6, K76.7
HIV/AIDS	**ICD-10:** B20, B97.35, Z21<br>**ATC:** J05AE01 , J05AE03, J05AE04, J05AE05, J05AE07, J05AE08, J05AE09, J05AE10, J05AF01, J05AF02, J05AF03, J05AF04, J05AF06, J05AF09, J05AF11, J05AF12, J05AG, J05AJ, J05AR, J05AX07, J05AX09, J05AX29
Impaired mobility	**ICD-10:** Z74.01, Z74.09, Z99.3<br>**Medical procedure code:** 520015, 520026, 520030, 520041, 520052, 520063, 520074, 520085, 520096, 520100, 520111, 520122, 520133, 520144, 520155, 520166, 520170, 520181, 520192, 520203, 520214, 520225, 520310, 520321, 520332, 520343, 520354, 520365, 520376, 520380, 520391, 520402, 520413, 520424, 520435, 520446, 520450, 520461, 520472, 520483, 520494, 520505, 520516, 520520, 520531, 520542, 520553, 520564, 520575, 520586, 520590, 520601, 520612, 520623, 520634, 520645, 520656, 520660, 520671, 520682, 520693, 520704, 520715,

	520726, 520730, 520741, 520752, 520763, 520774, 520785, 520796, 520800, 520811, 520822, 520833, 520844, 520855, 520866, 520870, 520881, 520892, 520903, 520914, 520925, 520936, 520940, 520951, 520962, 520973, 520984, 520995, 521006, 521010, 521021, 521032, 521043, 521054, 521065, 521076, 521080, 521091, 521102, 521113, 521124, 521135, 521146, 521150, 521161, 521172, 521183, 521194, 521205, 521216, 521220, 521231, 521242, 521253, 521264, 521275, 521286, 521290, 521301, 521312, 521323, 521334, 521345, 521356, 521360, 521371, 521382, 521393, 521404, 521415, 521426, 521430, 521441, 521452, 521463, 521474, 521485, 521496, 521500, 521511, 521522, 521533, 521544, 521555, 521566, 521570, 521581, 521592, 521603, 521614, 521625, 521636, 521640, 521651, 521662, 521673, 521684, 521695, 521706, 521710, 521721, 521732, 521743, 521754, 521765, 521776, 521780, 521791, 521802, 521813, 521824, 521835, 521846, 521850, 521861, 521872, 521883, 521894, 521905, 521916, 521920, 521931, 521942, 521953, 521964, 521975, 521986, 521990, 522001, 522012, 522023, 522034, 522045, 522056, 522060, 522071, 522082, 522093, 522104, 522115, 522126, 522130, 522141, 522152, 522163, 522174, 522185, 522196, 522200, 522211, 522222, 522233, 522244, 522255, 522266, 522270, 522281, 522292, 522303, 522314, 522325, 522336, 522340, 522351, 522362, 522373, 522384, 522395, 522406, 522410, 522421, 522432, 522443, 522454, 522465, 522476, 522480, 522535, 522550, 522572, 522583, 522594, 522605, 522616, 522620, 522631, 522642, 522653, 522664, 522675, 522686, 522734, 522745, 522756, 522760, 522771, 522782, 522793, 522804, 522815, 522826, 522830, 522841, 522852, 522863, 522874, 522885, 522896, 522900, 522911, 522922, 522933, 522944, 522955, 522966, 522970, 522981, 523014, 523025, 523036, 523040, 523051, 523062, 523073, 523084, 523095, 523106, 523110, 523121, 523132, 523143, 523154, 523165, 523176, 523180, 523191, 523202, 523213, 523224, 523235, 523246, 523250, 523261, 523272, 523283, 523294, 523305, 523316, 523320, 523331, 523342, 523353, 523364, 523375, 523386, 523390, 523401, 523412, 523423, 523434, 523445, 523456, 523460, 523471, 523482, 523493, 523504, 523515, 523526, 523530, 523541, 523552, 523563, 523574, 523585, 523596, 523600, 523611, 523622, 523633, 523644, 523655, 523666, 523670, 523681, 523692, 523703, 523714, 523725, 523736, 523740, 523751, 523762, 523773, 523784, 523795, 523806, 523810, 523821, 523832, 523843, 523854, 523865, 523876, 523880, 523891, 523902, 523913, 523924, 523935, 523946, 523950, 523961, 523972, 523983, 523994, 524005, 524016, 524020, 524031, 524042, 524053, 524064, 524075, 524086, 524090, 524101, 643451, 643462, 653656, 653660, 770394, 770405, 770416, 770420
Depression	**ICD-10:** F06.31, F06.32, F30, F31, F32, F33, F34.1, F43.21, F43.23, F53.0<br>**ATC**: N06AA, N06AB, N06AF, N06AG, N06AX01, N06AX02, N06AX03, N06AX04, N06AX05, N06AX06, N06AX07, N06AX08, N06AX09, N06AX10, N06AX11, N06AX13, N06AX14, N06AX15, N06AX16, N06AX17, N06AX18, N06AX19, N06AX21, N06AX22, N06AX23, N06AX24, N06AX25, N06AX26, N06AX27, N06AX28, N06AX29
Parkinson's disease	**ICD-10:** G20, G21, G23.1, G31.83, G31.85, G90.3<br>**ATC:** N04AB, N04AC, N04B
Arthritis	**ICD-10:** L40.5, M02.3, M05, M06, M08, M13.0, M13.1, M15-M19, M45, M46.1, M46.8, M46.9<br>**ATC**: L04AA13, L04AA24<br>**Medical procedure code:** 478030, 478041
Cognitive deterioration	**ICD-10:** G31.1, G31.84, G31.89, G31.9, R41.81
Paranoia	**ICD-10:** F06.0, F06.2, F20, F22, F23, F24, F28, F29
Chronic skin ulcer	**ICD-10:** E08.621, E08.622, E09.621, E09.622, E10.621, E10.622, E11.621, E11.622, E13.621, E13.622, L89, L97, L98.4<br>**Medical procedure code:** 114074, 114085
Skin and soft tissue infection	**ICD-10:** A06.7, A28.1, A31.1, A43.1, A46, A50.06, A51.3, A60.1, A63.0, A66.2, L00, L01, L02, L03, L04, L05, L08, L88<br>**Medical procedure codes:** 145552, 145563, 145574, 145585, 220253, 220264, 244650, 244661
Mycosis	**ICD-10:** B35-B49<br>**ATC:** D01A, D01BA02
Gout or other crystal-induced arthropathy	**ICD-10:** M10, M11, M1A<br>**ATC:** M04A
History of falling	**ICD-10:** R29.6, V00.141, V00.811, V00.831, V81.5, V81.6, V82.5, V82.6, V92.0, V93.3, V94.0, V97.0, W00, W01, W03, W05-W15, W16.0-W16.4, W17, W18, W19, Y21.1, Y21.3, Y30, Z91.81
Musculoskeletal problems	**ICD-10:** M02, M07, M12.0, M12.1, M12.2, M12.3, M12.4, M12.8, M12.9, M13, M14, M24.0, M24.3, M24.6, M24.7, M24.8, M24.9, M25, M36.1, M36.2, M36.3, M36.4, M45, M46.0, M46.1, M46.4, M46.8, M46.9, M47, M48, M49, M50, M51, M53, M54, M80, M81, M84.3, M84.4, M84.5, M84.6, Z87.31, Z87.39

Urinary tract infection	**ICD-10:** A56.01, N10, N12, N13.6, N15.1, N15.9, N16, N28.84, N28.85, N28.86, N30.0, N30.8, N30.9, N34, N39.0<br>**ATC:** J01XE01, J01XX01
**CLINICAL RISK SCORE**	
Charlson Comorbidity Index [6-10]	- **Myocardial infarction:** 1 point (definition mentioned above: 'Myocardial infarction')<br>- **Congestive heart failure**: 1 point (definition mentioned above: 'Congestive heart failure')<br>- **Peripheral vascular disease**: 1 point (definition mentioned above: 'Peripheral vascular disease)<br>- **Cerebrovascular disease:** 1 point (definition mentioned above: 'Cerebrovascular disease')<br>- **Dementia**: 1 point (definition mentioned above: 'Dementia')<br>- **Connective tissue disease**: 1 point (definition mentioned above: 'Connective tissue disease')<br>- **Peptic ulcer disease**: 1 point (definition mentioned above: 'Peptic ulcer disease')<br>- **Mild liver disease**: 1 point (definition mentioned above: 'Mild liver disease')<br>- **Diabetes without chronic complications**: 1 point (definition mentioned above: 'Diabetes mellitus', excluding patients with 'diabetes with chronic complications')<br>- **Diabetes with chronic complications**: 2 points (definition mentioned above: 'Diabetes with vascular complications')<br>- **Hemiplegia or paraplegia**: 2 points (definition mentioned above: 'Hemiplegia/paraplegia)<br>- **Renal disease:** 2 points (definition mentioned above: 'Chronic kidney disease')<br>- **Any malignancy, including leukaemia and lymphoma:** 2 points (definition mentioned above: 'Any malignancy, including leukaemia and lymphoma')<br>- **Moderate or severe liver disease:** 3 points (definition mentioned above: 'Moderate or severe liver disease')<br>- **Metastatic solid tumour:** 6 points (definition mentioned above: 'Metastatic cancer')<br>- **AIDS/HIV:** 6 points (definition mentioned above: 'HIV')<br>- **Age on the index date:**<br>• <50 years: 0 points<br>• 50-59 years: 1 point<br>• 60-69 years: 2 points<br>• 70-79 years: 3 points<br>• ≥80 years: 4 points<br>*The following comorbid conditions were mutually exclusive: diabetes with chronic complications and diabetes mellitus; mild liver disease and moderate or severe liver disease; and any malignancy and metastatic solid tumour*.
John Hopkins Claims-based Frailty Indicator [11-15]	- **Impaired mobility**: beta coefficient 1.24 (definition mentioned above: 'Impaired mobility')<br>- **Depression**: beta coefficient 0.54 (definition mentioned above: 'Depression')<br>- **Congestive heart failure**: beta coefficient 0.50 (definition mentioned above: 'Congestive heart failure')<br>- **Parkinson's disease**: beta coefficient 0.50 (definition mentioned above: 'Parkinson's disease')<br>- **White race**: beta coefficient -0.49: not available<br>- **Arthritis (any type):** beta coefficient 0.43 (definition mentioned above: 'Arthritis')<br>- **Cognitive impairment**: beta coefficient 0.33 (combination of definitions mentioned above: 'Dementia' and 'Cognitive deterioration')<br>• **ICD-10:** A81.0, F01, F02, F03, F05, G30, G31.0, G31.1, G31.83, G31.84, G31.85, G31.89, G31.9, R41.81<br>• **ATC:** N06D<br>- **Charlson comorbidity index (> 0)**: beta coefficient 0.31<br>- **Stroke**: beta coefficient 0.28 (definition mentioned above: 'Stroke')<br>- **Paranoia**: beta coefficient 0.24 (definition mentioned above: 'Schizophrenia & paranoia')<br>- **Chronic skin ulcer**: beta coefficient 0.23 (definition mentioned above: 'Chronic skin ulcer')<br>- **Pneumonia**: beta coefficient 0.21<br>• **ICD-10:** A01.03, A02.22, A37.01, A37.11, A37.81, A37.91, A50.04, A54.84, B01.2, B05.2, B06.81, B77.81, J09.X1, J09.X2, J09.X3, J10.0, J11.0, J12, J13, J14, J15, J16, J17, J18, J84.11, J84.2, J85.1, J95.851, Z87.01<br>- **Male sex**: beta coefficient -0.19<br>- **Skin and soft tissue infection**: beta coefficient 0.18 (definition mentioned above: 'Skin and soft tissue infection')<br>- **Mycoses**: beta coefficient 0.14 (definition mentioned above: 'Mycosis')<br>- **Age (for every 1 year increase)**: beta coefficient 0.09

	- **Admission in past 6 months**: beta coefficient 0.09<br>- **Gout or other crystal-induced arthropathy**: beta coefficient 0.08 (definition mentioned above: 'Gout or other crystal-induced arthropathy')<br>- **Falls**: beta coefficient 0.08 (definition mentioned above: 'History of falling')<br>- **Musculoskeletal problems**: beta coefficient 0.05 (definition mentioned above: 'Musculoskeletal problems')<br>- **Urinary tract infection**: beta coefficient 0.05 (definition mentioned above: 'Urinary tract infection')
**MEDICATION USE (≤1 year before follow-up)** [2]	
Maintenance therapy	**ATC:** R03<br><br>Categorized as:<br>*Combination therapy of long-acting β2-agonists (ATC: R03AC12, R03AC13, R03AC18, R03AC19) and inhaled corticosteroids (ATC: R03BA01, R03BA02, R03BA05) ~ due to use of the ATC for the specific categories or use of ATC: R03AK06 – R03AK08, R03AK10 R03AK12, R03AK14*<br>*Combination therapy of long-acting β2-agonists, long-acting muscarinic antagonists (ATC: R03BB04, R03BB05, R03BB06, R03BB07) and inhaled corticosteroids ~ due to use of the ATC for the specific categories or use of ATC: R03AL08, R03AL09, R03AL11, R03AL12*<br>*Other therapy: R03BA01, R03BA02, R03BA05, R03BC01, R03CC02, R03CC03, R03DA01, R03DA04, R03DC01, R03DC03, R03DC53*
Use of short-acting bronchodilators (SABD)	**ATC**: R03AC02, R03AC03, R03AL01, R03AL02, R03BB01<br>Categorized as appropriate (0-2 canisters/year), overuse (3-5 canisters/year) or heavy overuse (≥6 canisters/year)
Use of oral corticosteroids	**ATC:** H02AB04 (without parenteral products)
Antihistaminergic agents	**ATC:** R06, R01AC02, R01AC03, R01AC08
**OUTCOMES**	
Arrythmia	**ICD-10:** I44, I45, I46, I47, I48, I49<br>**Medical procedure code:** 180272, 180283, 180294, 180305, 589514, 589525, 589551, 589562
Congestive heart failure	**ICD-10:** I09.81, I11.0, I13.0, I13.2, I25.5, I42.0, I42.6-I42.9, I43, I50
Myocardial infarction	**ICD-10:** I21, I22
Peripheral vascular disease	**ICD-10:** I70, I71, I73.1, I73.8, I73.9, I74, I77, I79.0, K55.1, K55.8, K55.9<br>**Medical procedure code:** 229294, 229305, 229316, 229320, 229331, 229342, 235071, 235082, 235093, 235104, 235115, 235126, 235196, 235200, 235211, 235222, 236014, 236025, 236036, 236040, 236051, 236062, 237016, 237020, 237031, 237042, 237053, 237064, 237075, 237086, 237090, 237101, 237171, 237182, 589050, 589061, 589094, 589105, 589175, 589186, 589595, 589606, 589610, 589621, 589632, 589643, 589654, 589665
Pulmonary embolism	**ICD-10:** I26
Stroke	**ICD-10:** I61, I62, I63, I67.89, I69.1, I69.2, I69.3, I69.8, I69.9, I97.82<br>**Medical procedure code:** 182136, 182140, 182151, 182162, 182173, 182184, 477724, 477746, 477761, 477783
Mortality	All-cause mortality

eTable 3: Baseline characteristics of the study population with SMD before and after weighting.

A) Anti-IgE therapy

**Patient characteristics**	**No biologics n = 167,641**	**Anti-IgE n = 1,826**	**SMD before weighting**	**SMD after weighting**
Age in years (SD)	63.9 (17.2)	53.7 (16.3)	**0.624**	0.013
Female	92,814 (55.4%)	1,039 (56.9%)	0.031	0.007
Low SES	57,330 (34.2%)	485 (26.6%)	**0.173**	0.008
Follow-up time in years (SD)	2.9 (1.5)	3.1 (1.5)		
Smoking status				
No smoker	108,853 (64.9%)	1,261 (69.1%)	0.089	0.003
Past smoker	41,219 (24.6%)	415 (22.7%)	0.044	0.005
Current smoker	17,569 (10.5%)	150 (8.2%)	0.083	0.004
Obesity or overweight	17,050 (10.2%)	177 (9.7%)	0.016	0.008
Exacerbation history				
Two or more exacerbations	57,253 (34.2%)	1,007 (55.1%)	**0.422**	0.015
**Comorbidities**				
Upper respiratory	45,543 (27.2%)	771 (42.2%)	**0.305**	0.025
Allergic skin disease	30,683 (18.3%)	406 (22.2%)	0.095	0.003
Upper GTD	12,176 (7.3%)	142 (7.8%)	0.019	0.003
Dyslipidaemia	63,995 (38.2%)	481 (26.3%)	**0.269**	0.010
Diabetes mellitus	32,890 (19.6%)	290 (15.9%)	**0.102**	0.012
Hypertension	62,053 (37.0%)	390 (21.4%)	**0.382**	0.008
Prior CV disease	43,403 (25.9%)	241 (13.2%)	**0.375**	0.004
Frailty	29,457 (17.6%)	54 (3.0%)	**0.863**	0.008
**Drug use**				
Maintenance therapy (beside biologics)				
ICS-LABA	103,776 (61.9%)	1,115 (61.1%)	0.017	0.017
ICS-LABA-LAMA	36,356 (21.7%)	488 (26.7%)	**0.114**	0.002
Other therapy	27,509 (16.4%)	223 (12.2%)	**0.128**	0.022
SABD use				
Appropriate (0-2 canisters/year)	107,887 (64.4%)	839 (45.9%)	**0.369**	0.024
Overuse (3-5 canisters/year)	28,321 (16.9%)	326 (17.9%)	0.025	0.006
Heavy overuse (≥6 canisters/year)	31,433 (18.8%)	661 (36.2%)	**0.363**	0.020
≥6 packages OCS	10,298 (6.1%)	182 (10.0%)	**0.128**	0.009
Antihistamines	60,989 (36.4%)	1,229 (67.3%)	**0.659**	0.010

SMDs values were calculated with the mean of the treated group minus the mean of the control group as numerator, and with the standard deviation of the treated group as the denominator. SMD values ≥0.1 are presented in bold, a threshold used to indicate imbalance between treatment groups before and after weighting.[16]

B) Anti-IL5/IL5R therapy

Patient characteristics	No biologics n = 167,641	Anti-IgE n = 1,826	SMD before weighting	SMD after weighting
Age in years (SD)	63.9 (17.2)	58.7 (14.5)	**0.357**	0.005
Female	92,814 (55.4%)	1,248 (52.0%)	0.067	0.013
Low SES	57,330 (34.2%)	644 (26.9%)	**0.166**	0.012
Follow-up time in years (SD)	2.9 (1.5)	2.6 (1.4)		
Smoking status				
No smoker	108,853 (64.9%)	1,521 (63.4%)	0.031	0.026
Past smoker	41,219 (24.6%)	716 (29.9%)	**0.115**	0.027
Current smoker	17,569 (10.5%)	161 (6.7%)	**0.151**	0.002
Obesity or overweight	17,050 (10.2%)	299 (12.5%)	0.070	0.007
Exacerbation history				
Two or more exacerbations	57,253 (34.2%)	1,699 (70.9%)	**0.808**	0.031
**Comorbidities**				
Upper respiratory	45,543 (27.2%)	1,167 (48.7%)	**0.430**	0.067
Allergic skin disease	30,683 (18.3%)	424 (17.7%)	0.016	0.001
Upper GTD	12,176 (7.3%)	232 (9.7%)	0.082	0.000
Dyslipidaemi**a**	63,995 (38.2%)	804 (33.5%)	0.098	0.011
Diabetes mellitus	32,890 (19.6%)	435 (18.1%)	0.038	0.003
Hypertension	62,053 (37.0%)	656 (27.4%)	**0.217**	0.005
Prior CV disease	43,403 (25.9%)	438 (18.3%)	**0.197**	0.010
Frailty	29,457 (17.6%)	102 (4.3%)	**0.660**	0.013
**Drug use**				
Maintenance therapy (beside biologics)				
ICS-LABA	103,776 (61.9%)	1,504 (62.7%)	0.017	0.059
ICS-LABA-LAMA	36,356 (21.7%)	859 (35.8%)	0.295	0.053
Other therapy	27,509 (16.4%)	35 (1.5%)	**1.247**	0.028
SABD use				
Appropriate (0-2 canisters/year)	107,887 (64.4%)	960 (40.0%)	**0.496**	0.036
Overuse (3-5 canisters/year)	28,321 (16.9%)	450 (18.8%)	0.048	0.013
Heavy overuse (≥6 canisters/year)	31,433 (18.8%)	988 (41.2%)	**0.456**	0.046
≥6 packages OCS	10,298 (6.1%)	415 (17.3%)	**0.295**	0.029
Antihistamines	60,989 (36.4%)	1,232 (51.4%)	**0.300**	0.021

SMDs values were calculated with the mean of the treated group minus the mean of the control group as numerator, and with the standard deviation of the treated group as the denominator. SMD values ≥0.1 are presented in bold, a threshold used to indicate imbalance between treatment groups before and after weighting.[16]

eTable 4: Crude event rates per outcome stratified by treatment.

Outcome	No biologics n = 167,641	Anti-IgE n = 1,826	Anti-IL5/IL5R n = 2,398
Myocardial infarction			
Number of events	3,908	30	38
Event rate per 100 PYs (95% CI)	0.82 (0.79-0.84)	0.55 (0.37-0.78)	0.62 (0.44-0.86)
Congestive heart failure			
Number of events	22,662	117	143
Event rate per 100 PYs (95% CI)	5.01 (4.94-5.08)	2.18 (1.81-2.62)	2.40 (2.03-2.83)
Arrythmia			
Number of events	30,189	178	242
Event rate per 100 PYs (95% CI)	6.87 (6.79-6.95)	3.38 (2.90-3.92)	4.19 (3.68-4.76)
Peripheral vascular disease			
Number of events	11,622	59	88
Event rate per 100 PYs (95% CI)	2.49 (2.45-2.54)	1.08 (0.82-1.39)	1.46 (1.17-1.80)
Pulmonary embolism			
Number of events	2,950	26	29
Event rate per 100 PYs (95% CI)	0.61 (0.59-0.64)	0.47 (0.31-0.69)	0.47 (0.32-0.68)
Stroke			
Number of events	6,553	24	59
Event rate per 100 PYs (95% CI)	1.38 (1.35-1.41)	0.43 (0.28-0.65)	0.97 (0.74-1.25)
Mortality			
Number of events	35,712	118	126
Event rate per 100 PYs (95% CI)	7.38 (7.30-7.45)	2.12 (1.76-2.54)	2.05 (1.71-2.44)

eTable 4: The number of events and crude event rates per 100 person-years (PYs) and the 95% confidence interval of outcomes, stratified by treatment.

eTable 5: Sensitivity analysis – aHR of outcomes after IPTW in patients with incident biologic use.

Outcome	Anti-IgE (n = 749) aHR (95% CI)	P-value	Anti-IL5/IL5R (n = 1,704) aHR (95% CI)	P-value
Myocardial infarction	1.18 (0.64-2.17)	0.599	0.77 (0.50-1.19)	0.234
Congestive heart failure	0.83 (0.60-1.15)	0.269	0.65 (0.52-0.81)	<0.001
Arrythmia	0.76 (0.57-1.01)	0.058	0.75 (0.63-0.89)	<0.001
Peripheral vascular disease	0.49 (0.28-0.84)	0.010	0.73 (0.55-0.97)	0.029
Pulmonary embolism	1.22 (0.59-2.54)	0.597	0.89 (0.53-1.49)	0.662
Stroke	0.74 (0.38-1.44)	0.379	1.17 (0.84-1.63)	0.345
Mortality	0.44 (0.31-0.63)	<0.001	0.33 (0.26-0.42)	<0.001

eTable 5: Hazard ratios of outcomes compared between asthma patients initiated with anti-IgE versus non-biological therapies, and compared between asthma patients initiated with anti-IL5/IL5R versus non-biological therapies after IPTW.

eTable 6: Sensitivity analysis – aHR of outcomes after IPTW in patients without COPD disease label or aged ≤60 years.

Outcome	Anti-IgE<br>No COPD label<br>aHR (95% CI)	 ≤60 years<br>aHR (95% CI)	Anti-IL5/IL5R<br>No COPD label<br>aHR (95% CI)	 ≤60 years<br>aHR (95% CI)
Myocardial infarction	1.09 (0.72-1.65)	1.19 (0.65-2.19)	0.89 (0.58-1.36)	0.64 (0.29-1.42)
Congestive heart failure	**0.68 (0.53-0.87)**	0.84 (0.58-1.24)	**0.58 (0.45-0.75)**	0.78 (0.53-1.16)
Arrythmia	**0.78 (0.64-0.94)**	0.87 (0.64-1.19)	**0.78 (0.65-0.93)**	0.84 (0.61-1.17)
Peripheral vascular disease	**0.64 (0.46-0.88)**	0.71 (0.43-1.20)	**0.60 (0.44-0.84)**	0.90 (0.55-1.47)
Pulmonary embolism	0.89 (0.55-1.44)	1.45 (0.82-2.55)	**0.55 (0.31-0.98)**	**0.22 (0.05-0.93)**
Stroke	**0.35 (0.20-0.62)**	0.56 (0.28-1.14)	0.89 (0.61-1.28)	0.82 (0.45-1.52)
Mortality	**0.37 (0.29-0.49)**	**0.32 (0.20-0.50)**	**0.27 (0.20-0.36)**	**0.32 (0.21-0.50)**
Significant results (p < 0.05) are presented in bold.				

eTable 6: Hazard ratios of outcomes compared between asthma patients without a COPD disease label or aged ≤60 years treated with anti-IgE versus non-biological therapies, and compared between asthma patients without a COPD disease label or aged ≤60 years treated with anti-IL5/IL5R versus non-biological therapies after IPTW.

eTable 7 Sensitivity analysis – aHR of outcomes after IPTW in patients with inclusion between January, 2017 and March, 2020.

Outcome	Anti-IgE (n = 1,462)<br>aHR (95% CI)	 P-value	Anti-IL5/IL5R (n = 1,698)<br>aHR (95% CI)	 P-value
Myocardial infarction	1.00 (0.70-1.45)	0.980	0.79 (0.54-1.14)	0.208
Congestive heart failure	0.83 (0.69-1.00)	0.051	0.69 (0.57-0.83)	<0.001
Arrythmia	0.89 (0.76-1.04)	0.135	0.83 (0.72-0.96)	0.014
Peripheral vascular disease	0.68 (0.53-0.89)	0.005	0.68 (0.53-0.87)	0.002
Pulmonary embolism	1.10 (0.75-1.63)	0.621	0.85 (0.55-1.30)	0.444
Stroke	0.58 (0.39-0.88)	0.009	0.94 (0.69-1.27)	0.688
Mortality	0.50 (0.41-0.60)	<0.001	0.36 (0.29-0.44)	<0.001

eTable 7: Hazard ratios of outcomes compared between asthma patients treated with anti-IgE versus non-biological therapies (between January 2017 and March, 2020), and compared between asthma patients treated with anti-IL5/IL5R versus non-biological therapies (between January 2017 and March, 2020) after IPTW.

eTable 8: Sensitivity analysis – HR of outcomes after PSM.

Outcome	Anti-IgE (n = 1,826 per group)<br>HR (95% CI)	 P-value	Anti-IL5/IL5R (n = 2,398 per group)<br>HR (95% CI)	 P-value
Myocardial infarction	0.80 (0.50-1.30)	0.372	0.65 (0.44-0.97)	0.036
Congestive heart failure	0.77 (0.61-0.98)	0.031	0.59 (0.48-0.72)	<0.001
Arrythmia	0.89 (0.73-1.09)	0.258	0.71 (0.61-0.84)	<0.001
Peripheral vascular disease	0.91 (0.64-1.28)	0.581	0.58 (0.44-0.74)	<0.001
Pulmonary embolism	0.73 (0.44-1.21)	0.219	0.66 (0.42-1.04)	0.074
Stroke	0.55 (0.33-0.90)	0.016	1.31 (0.90-1.89)	0.159
Mortality	0.49 (0.40-0.61)	<0.001	0.33 (0.27-0.40)	<0.001

eTable 8: Hazard ratios of outcomes compared between asthma patients treated with anti-IgE versus non-biological therapies, and compared between asthma patients treated with anti-IL5/IL5R versus non-biological therapies after propensity score matching (PSM).

eTable 9: Sensitivity analysis – aOR of outcomes (nested case-control analysis)

Outcome	Anti-IgE aOR (95% CI)	Anti-IgE P-value	Anti-IL5/IL5R aOR (95% CI)	Anti-IL5/IL5R P-value
Myocardial infarction	1.04 (0.69-1.59)	0.843	1.04 (0.69-1.59)	0.843
Congestive heart failure	0.86 (0.69-1.07)	0.166	0.86 (0.69-1.07)	0.166
Arrythmia	0.89 (0.75-1.06)	0.195	0.89 (0.75-1.06)	0.195
Peripheral vascular disease	0.72 (0.54-0.96)	0.027	0.72 (0.54-0.96)	0.027
Pulmonary embolism	0.81 (0.52-1.26)	0.352	0.81 (0.52-1.26)	0.352
Stroke	0.58 (0.38-0.90)	0.016	0.58 (0.38-0.90)	0.016
Mortality	0.52 (0.42-0.63)	<0.001	0.52 (0.42-0.63)	<0.001

eTable 9: Adjusted odds ratios of the association between biological use, and cardiovascular outcomes and mortality using a nested case-control design. The analysis were also adjusted for SES, obesity or overweight, smoking status, dyslipidaemia, diabetes mellitus, upper respiratory comorbidities, allergic skin disease, upper gastro-intestinal tract disorder, hypertension, history of CV disease, frailty, exacerbation history, maintenance therapy, SABD use, OCS use and antihistamine use.

eTable 10: Sensitivity analysis – aHR of outcomes after IPTW, stratified by treatment response

Outcome	Anti-IgE Responder aHR (95% CI)	Anti-IgE No responder aHR (95% CI)	Anti-IL5/IL5R Responder aHR (95% CI)	Anti-IL5/IL5R No responder aHR (95% CI)
Myocardial infarction	1.04 (0.63-1.72)	1.02 (0.59-1.73)	1.02 (0.64-1.63)	0.60 (0.33-1.08)
Congestive heart failure	**0.71 (0.54-0.94)**	0.87 (0.66-1.14)	**0.70 (0.55-0.90)**	**0.65 (0.48-0.86)**
Arrythmia	0.83 (0.67-1.04)	0.94 (0.74-1.18)	0.94 (0.78-1.12)	**0.71 (0.56-0.91)**
Peripheral vascular disease	**0.61 (0.41-0.90)**	0.74 (0.50-1.08)	**0.64 (0.46-0.89)**	0.80 (0.56-1.13)
Pulmonary embolism	1.04 (0.58-1.87)	1.05 (0.60-1.83)	0.84 (0.47-1.48)	0.62 (0.30-1.29)
Stroke	**0.45 (0.25-0.82)**	0.61 (0.33-1.14)	1.10 (0.76-1.60)	0.96 (0.58-1.59)
Mortality	**0.51 (0.36-0.70)**	**0.52 (0.38-0.70)**	**0.45 (0.33-0.62)**	**0.39 (0.28-0.55)**
Significant results ($p < 0.05$) are presented in bold.				

eTable 10: Hazard ratios of outcomes compared between asthma patients treated with anti-IgE versus non-biological therapies, and compared between asthma patients treated with anti-IL5/IL5R versus non-biological therapies stratified by treatment response after IPTW.

eTable 11: Sensitivity analysis – aOR of outcomes (nested case-control analysis with interaction terms)

A) Anti-IgE therapy

	MI aOR (95% CI)	Heart failure aOR (95% CI)	Arrythmia aOR (95% CI)	PAD aOR (95% CI)	PE aOR (95% CI)	Stroke aOR (95% CI)	Mortality aOR (95% CI)
Anti-IgE use	1.65 (0.90-3.04)	1.02 (0.74-1.41)	0.96 (0.74-1.23)	0.68 (0.43-1.10)	1.17 (0.61-2.26)	**0.42 (0.19-0.92)**	**0.70 (0.50-0.98)**
Hospitalised exacerbation	**1.41 (1.25-1.59)**	**1.27 (1.19-1.35)**	**1.11 (1.05-1.18)**	**1.02 (0.95-1.11)**	**1.81 (1.57-2.09)**	**1.04 (0.93-1.15)**	**2.13 (2.05-2.21)**
SABD overuse	**1.27 (1.15-1.41)**	**1.54 (1.48-1.61)**	**1.34 (1.29-1.39)**	**1.23 (1.16-1.31)**	**1.51 (1.35-1.69)**	**1.12 (1.04-1.21)**	**1.62 (1.57-1.68)**
Initiated CV drug	**1.64 (1.45-1.85)**	**2.77 (2.63-2.93)**	**2.68 (2.57-2.79)**	**2.38 (2.21-2.57)**	**1.93 (1.68-2.21)**	**1.94 (1.77-2.14)**	**3.63 (3.49-3.78)**
High OCS use	**1.60 (1.37-1.86)**	**1.60 (1.50-1.71)**	**1.54 (1.46-1.63)**	**1.54 (1.41-1.68)**	**2.16 (1.84-2.55)**	**1.34 (1.18-1.51)**	**1.67 (1.59-1.76)**
Anti-IgE x hosp. exac.	2.73 (0.70-10.58)	1.44 (0.78-2.68)	1.69 (1.00-2.88)	**2.77 (1.25-6.16)**	1.12 (0.29-4.33)	**5.40 (1.63-17.90)**	1.32 (0.80-2.18)
Anti-IgE x SABD overuse	0.59 (0.23-1.53)	0.90 (0.55-1.45)	1.00 (0.68-1.45)	1.08 (0.55-2.13)	0.38 (0.13-1.14)	2.38 (0.89-6.34)	0.69 (0.44-1.09)
Anti-IgE x initiated CV drug	0.44 (0.17-1.13)	0.71 (0.43-1.16)	0.89 (0.61-1.30)	0.79 (0.42-1.49)	1.34 (0.53-3.38)	0.56 (0.20-1.52)	0.89 (0.58-1.37)
Anti-IgE x high OCS use	2.31 (0.53-10.03)	0.96 (0.47-1.94)	0.83 (0.46-1.49)	1.02 (0.44-2.37)	0.83 (0.16-4.36)	0.41 (0.08-2.13)	0.72 (0.41-1.30)

A) Anti-IL5/IL5R therapy

	MI aOR (95% CI)	Heart failure aOR (95% CI)	Arrythmia aOR (95% CI)	PAD aOR (95% CI)	PE aOR (95% CI)	Stroke aOR (95% CI)	Mortality aOR (95% CI)
Anti-IL5/IL5R use	0.91 (0.54-1.55)	0.99 (0.77-1.27)	0.97 (0.80-1.17)	**0.71 (0.51-0.98)**	1.53 (0.86-2.69)	1.28 (0.87-1.87)	**0.65 (0.50-0.85)**
Hospitalised exacerbation	**1.41 (1.25-1.59)**	**1.27 (1.19-1.35)**	**1.11 (1.05-1.18)**	**1.02 (0.95-1.11)**	**1.81 (1.57-2.09)**	**1.04 (0.93-1.15)**	**2.13 (2.05-2.21)**
SABD overuse	**1.27 (1.15- 1.41)**	**1.54 (1.48-1.61)**	**1.34 (1.29-1.39)**	**1.23 (1.16-1.31)**	**1.51 (1.35-1.69)**	**1.12 (1.04- 1.21)**	**1.62 (1.57-1.68)**
Initiated CV drug	**1.64 (1.45- 1.85)**	**2.77 (2.63-2.93)**	**2.68 (2.57-2.79)**	**2.38 (2.21-2.57)**	**1.93 (1.68-2.21)**	**1.94 (1.77- 2.14)**	**3.63 (3.49-3.78)**
High OCS use	**1.60 (1.37- 1.86)**	**1.60 (1.50-1.71)**	**1.54 (1.46-1.63)**	**1.54 (1.41-1.68)**	**2.16 (1.84-2.55)**	**1.34 (1.18- 1.51)**	**1.67 (1.59-1.76)**
Anti-IL5(R) x hosp.exac.	1.79 (0.57- 5.58)	1.75 (0.90-3.37)	0.69 (0.37-1.28)	**2.57 (1.12-5.92)**	0.24 (0.04-1.38)	1.20 (0.42- 3.42)	0.77 (0.44-1.35)
Anti-IL5(R) x SABD overuse	1.01 (0.42- 2.45)	**0.54 (0.33-0.90)**	1.26 (0.88-1.80)	0.82 (0.46-1.45)	1.51 (0.53-4.25)	1.29 (0.58- 2.90)	0.85 (0.53-1.35)
Anti-IL5(R)x initiated CV drug	0.64 (0.27- 1.54)	1.16 (0.73-1.84)	1.05 (0.74-1.51)	1.06 (0.61-1.85)	0.60 (0.21-1.68)	0.68 (0.33- 1.38)	0.75 (0.48-1.19)
Anti-IL5(R) x high OCS use	1.24 (0.34- 4.51)	0.60 (0.28-1.25)	0.65 (0.37-1.12)	0.76 (0.29-2.02)	1.02 (0.18-5.73)	0.40 (0.11- 1.51)	1.18 (0.65-2.14)

eTable 11: Adjusted odds ratios of the association between biological use, and cardiovascular outcomes and mortality using a nested case-control design. The analyses were also adjusted for SES, obesity or overweight, smoking status, dyslipidaemia, diabetes mellitus, upper respiratory comorbidities, allergic skin disease, upper gastro-intestinal tract disorder, hypertension, history of CV disease, frailty, exacerbation history, maintenance therapy, SABD use, OCS use and antihistamine use. Hospitalised exacerbations were defined as experiencing at least 1 hospitalised exacerbation, SABD overuse was defined as ≥3 dispensed canisters, frequent OCS use was defined as ≥6 filled prescriptions, newly initiated CVD management was defined as the use of CV medication (including lipid-lowering drugs) in those without prior CVD. Statistically significant ($p<0.05$) results are presented in bold. aOR: adjusted odds ratio, MI: myocardial infarction, SABD: short-acting bronchodilator, PAD: peripheral artery disease, PE: pulmonary embolism.

eTable 12: Coefficients from the PS model.

A) Anti-IgE therapy

Variable	2017	2018	2019	2020	2021
Age	-0.025	-0.027	-0.017	-0.026	-0.021
Female	-0.070	0.150	0.076	0.034	0.154
Low SES	-0.187	-0.529	-0.435	-0.118	-0.496
Past smoker	-0.320	-0.339	-0.373	-0.294	-0.247
Current smoker	-0.692	-0.305	-0.769	-0.295	-0.012
Obesity or overweight	0.351	0.053	0.032	0.367	0.020
Two or more exacerbations	0.958	0.905	1.030	0.518	0.888
Upper respiratory comorbidities	0.543	0.306	0.380	0.007	0.177
Allergic skin diseases	-0.087	0.377	0.019	0.069	0.368
Upper gastro-intestinal tract disorder	0.243	0.132	0.504	0.543	0.235
Dyslipidaemia	0.008	0.017	-0.058	-0.175	-0.142
Diabetes Mellitus	0.171	0.135	0.236	-0.365	-0.516
Hypertension	-0.304	-0.187	-0.405	-0.374	-0.351
Prior CV disease	-0.510	-0.428	-0.125	-0.009	-0.464
Frailty	-0.701	-0.523	-1.004	-0.895	-1.030
ICS-LABA-LAMA	0.224	0.714	0.361	0.613	1.005
Other therapy	-0.315	0.118	-0.586	0.265	0.807
SABD overuse	0.480	0.635	0.947	0.531	0.094
Heavy SABD overuse	0.926	1.442	1.535	1.403	0.957
High OCS use	0.377	-0.086	-0.323	0.625	0.135
Antihistamines	0.857	1.011	1.105	1.418	1.391

B) Anti-IL5/IL5R therapy

Variable	2017	2018	2019	2020	2021
Age	-0.019	-0.010	-0.010	-0.001	-0.008
Female	0.092	-0.082	0.089	-0.096	-0.003
Low SES	-0.260	-0.513	-0.526	-0.162	-0.616
Past smoker	-0.545	-0.285	-0.325	-0.122	0.017
Current smoker	-1.060	-0.884	-0.430	-1.141	-0.721
Obesity or overweight	0.327	0.301	0.511	0.289	0.186
Two or more exacerbations	1.498	1.465	1.408	1.399	1.310
Upper respiratory comorbidities	1.058	0.951	0.874	0.982	1.062
Allergic skin diseases	-0.184	-0.234	-0.030	0.092	-0.111
Upper gastro-intestinal tract disorder	0.363	0.450	0.295	0.158	0.509
Dyslipidaemia	-0.097	0.061	0.271	-0.168	0.014
Diabetes Mellitus	0.103	-0.037	-0.214	-0.008	-0.009
Hypertension	-0.282	-0.201	-0.254	-0.259	-0.342
Prior CV disease	-0.219	-0.535	-0.135	-0.287	-0.273
Frailty	-1.033	-0.797	-0.566	-0.727	-0.565
ICS-LABA-LAMA	0.020	0.326	0.341	0.388	0.767
Other therapy	-3.111	-2.590	-2.595	-1.724	-1.682
SABD overuse	0.438	1.110	0.895	0.674	0.926
Heavy SABD overuse	0.945	1.637	1.420	1.648	1.098
High OCS use	0.799	0.527	0.495	0.397	0.503
Antihistamines	0.197	0.325	0.530	0.415	0.567

Supplemental figures

eFigure 1: Flowchart of inclusion of study population

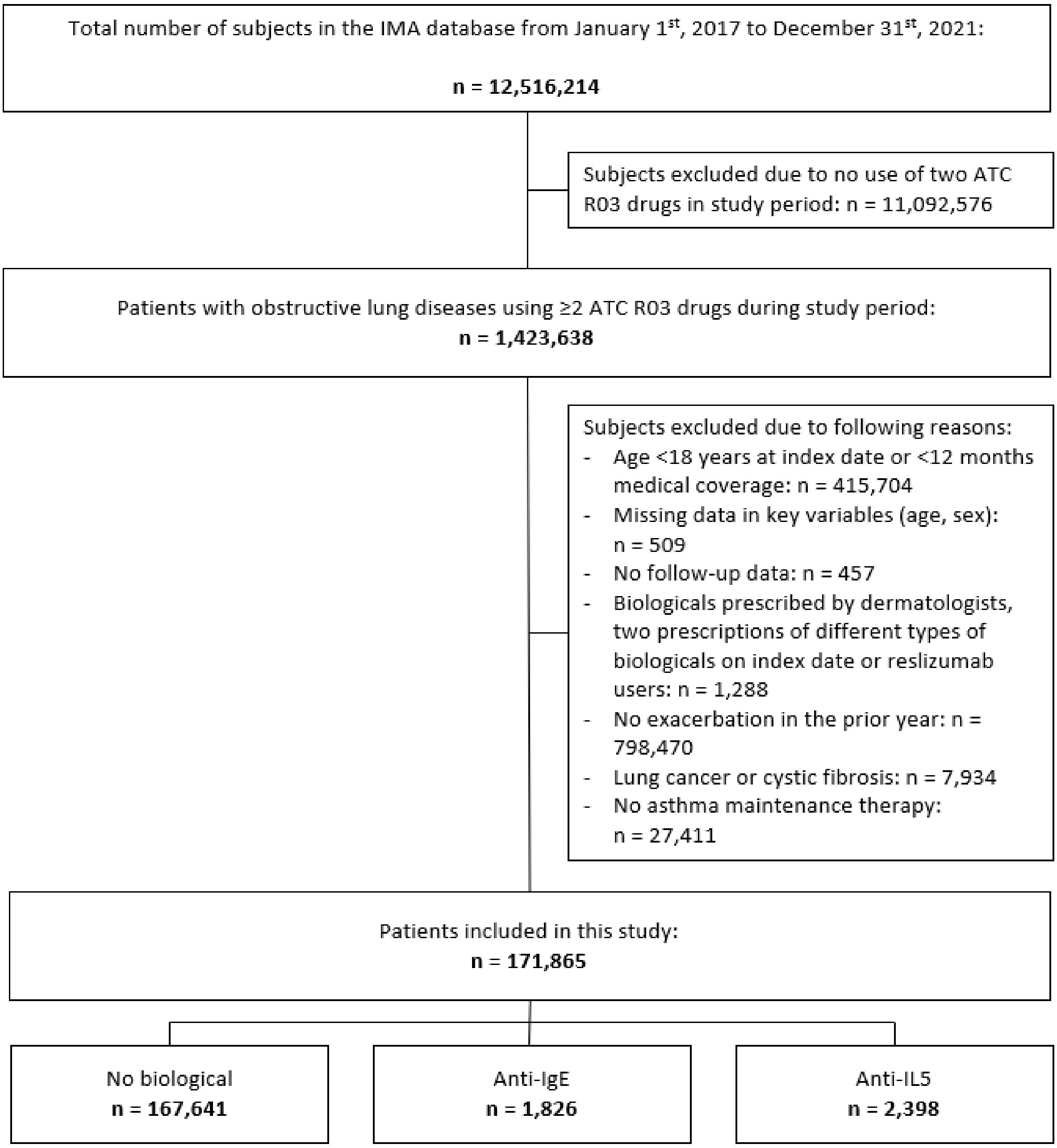

eFigure 1: Flowchart of inclusion of study population

eFigure 2: Love plot of propensity score model for anti-IgE use

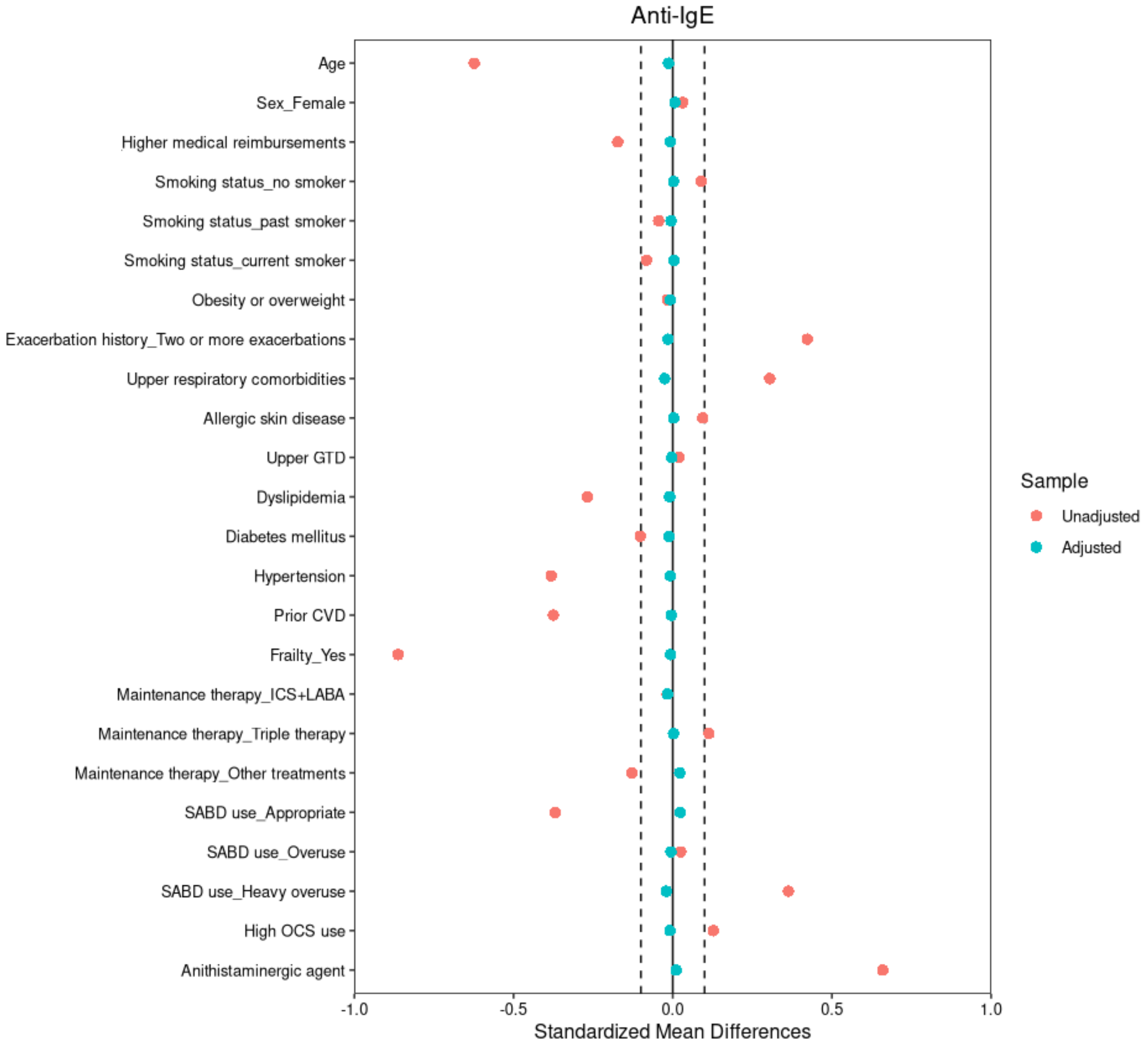

eFigure 2: Love plot of propensity score model for anti-IgE use.
Love plot illustrating balance before and after IPTW when comparing anti-IgE users to non-biological users. All covariates were balanced (standardized mean difference <0.1).

eFigure 3: Love plot of propensity score model for anti-IL5/IL5R use

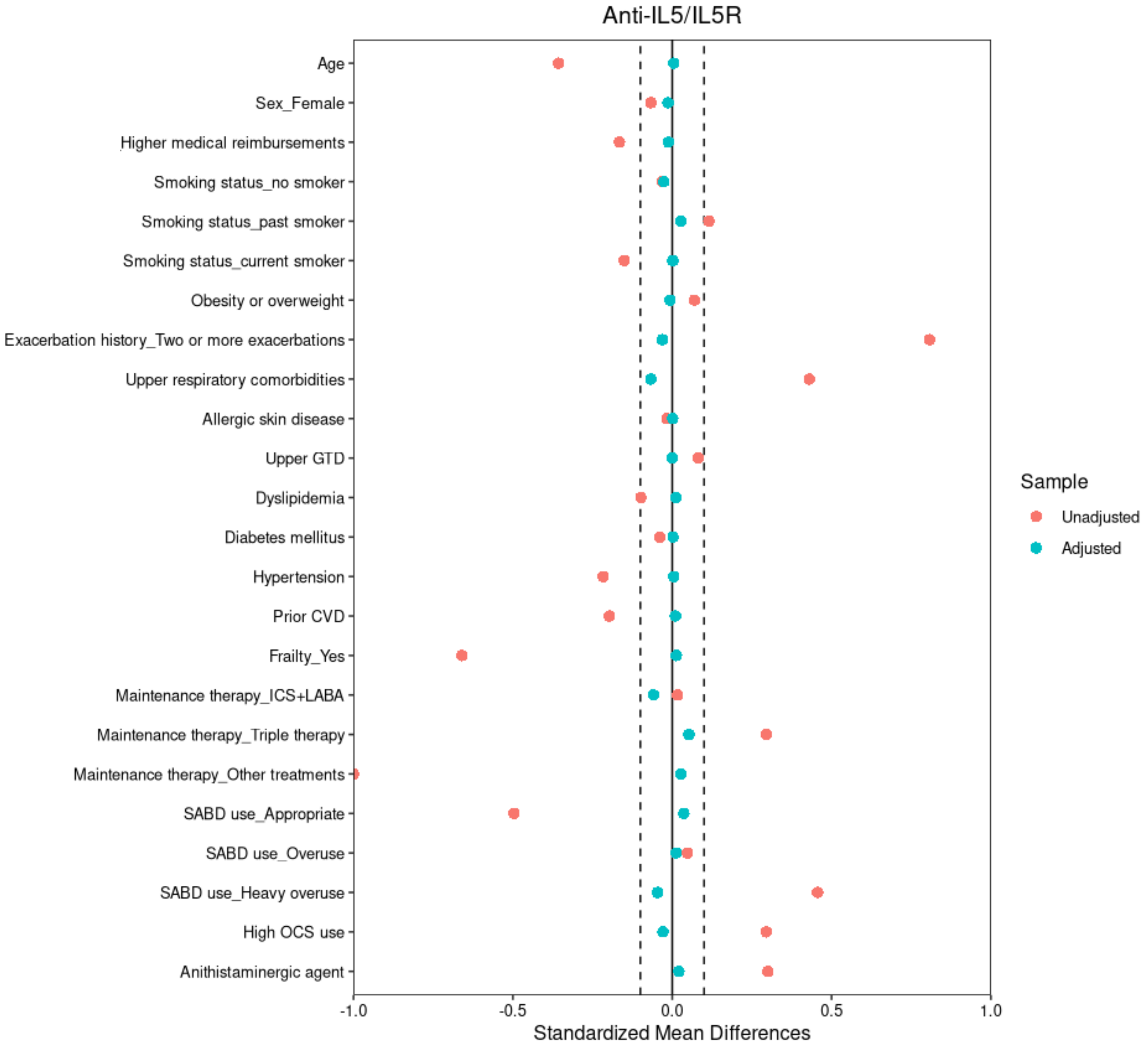

eFigure 3: Love plot of propensity score model for anti-IL5/IL5R use.
Love plot illustrating balance before and after IPTW when comparing anti-IL5/IL5R to non-biological users. All covariates were balanced (standardized mean difference <0.1).

eFigure 4: Mirrored histogram plot of propensity scores of anti-IgE therapy after adjustment.

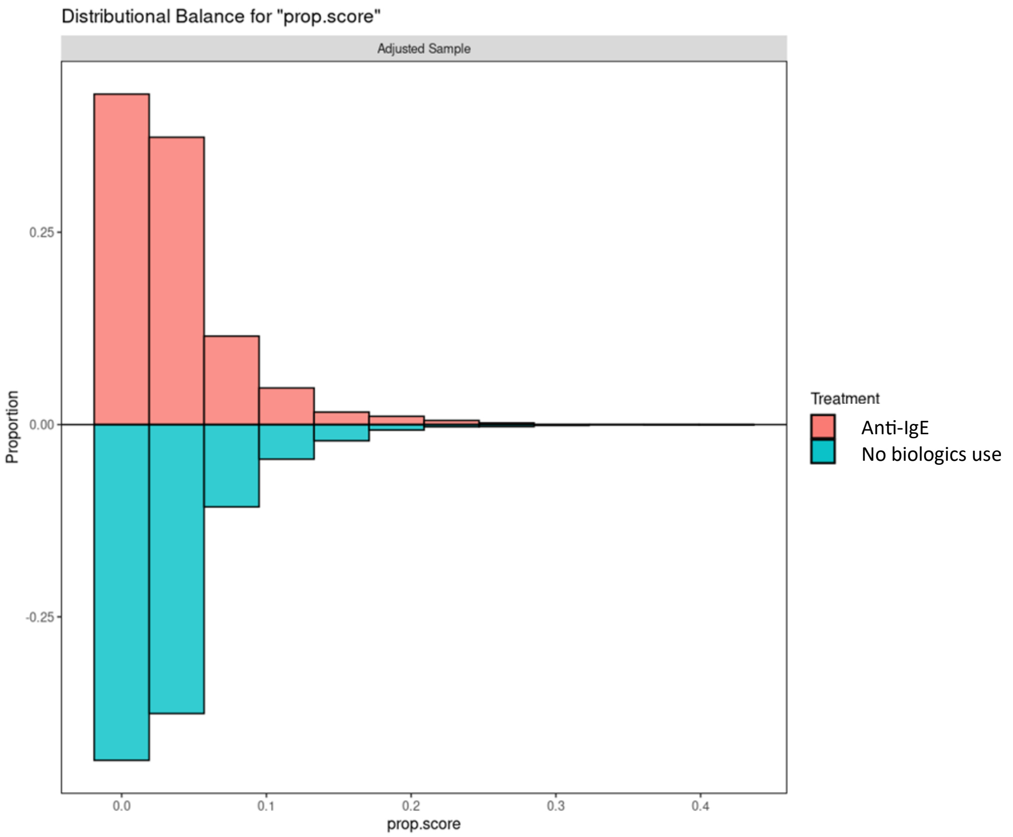

eFigure 5: Mirrored histogram plot of propensity scores of anti-IL5/IL5R therapy after adjustment.

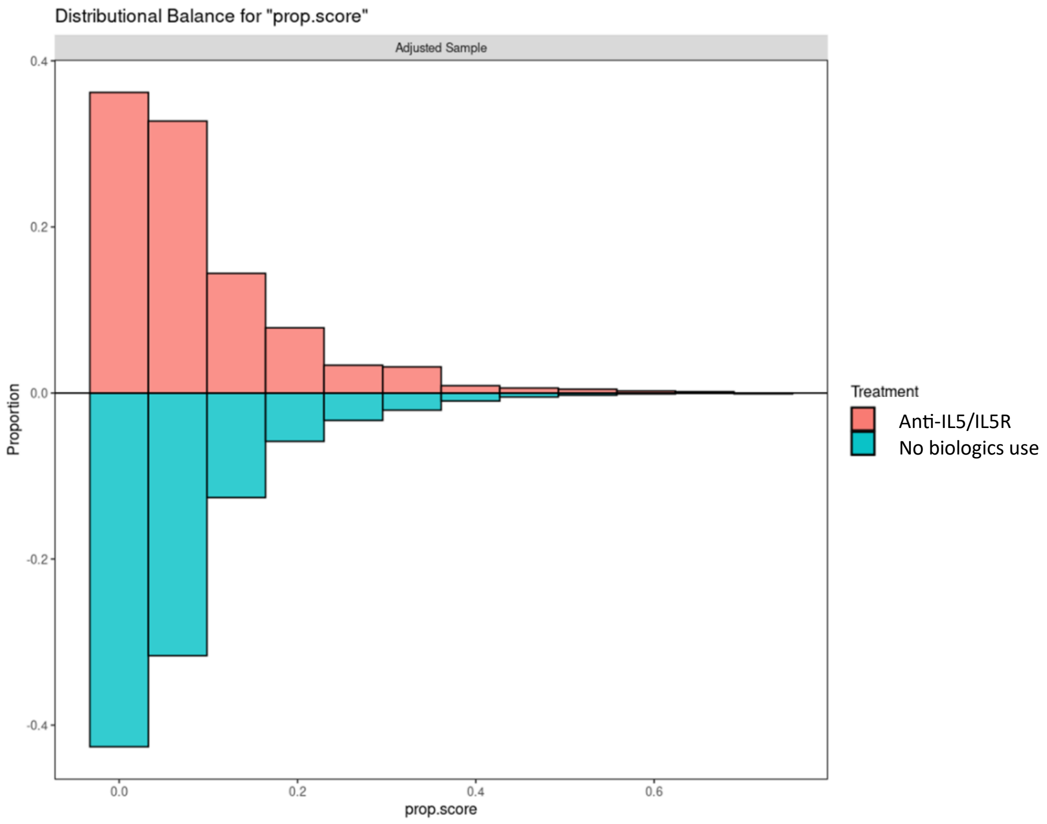

References

1. Von Elm E, Altman DG, Egger M, Pocock SJ, Gøtzsche PC, Vandenbroucke JP. The Strengthening the Reporting of Observational Studies in Epidemiology (STROBE) statement: guidelines for reporting observational studies. Lancet. 2007;370(9596):1453-7.

2. WHO Collaborating Centre for Drug Statistics Methodology. Available from: https://www.whocc.no/. Accessed 25 November 2021.

3. The International Classification of Diseases (ICD), Clinical Modification. Available from: https://www.cdc.gov/nchs/icd/index.htm. Accessed 25 November 2021.

4. RIZIV/INAMI (Rijksinstituut voor ziekte- en invaliditeitsverzekering/Institut national d'assurance maladie-invalidité) medical procedure codes for claims of ambulatory and hospital care. Available from: https://www.riziv.fgov.be/nl/nomenclatuur/Paginas/default.aspx (in Dutch/French). Accessed 25 November 2021.

5. Lorenzoni G, Baldi I, Soattin M, Gregori D, Buja A. A Systematic Review of Case-Identification Algorithms Based on Italian Healthcare Administrative Databases for Three Relevant Diseases of the Cardiovascular System: Hypertension, Heart Failure, and Congenital Heart Diseases. Epidemiol Prev. 2019;43(4 Suppl 2):51-61.

6. Quan H, Sundararajan V, Halfon P, et al. Coding algorithms for defining comorbidities in ICD-9-CM and ICD-10 administrative data. Med Care. 2005;43(11):1130-9.

7. Sundararajan V, Henderson T, Perry C, Muggivan A, Quan H, Ghali WA. New ICD-10 version of the Charlson comorbidity index predicted in-hospital mortality. J Clin Epidemiol. 2004;57(12):1288-94.

8. Charlson M, Szatrowski TP, Peterson J, Gold J. Validation of a combined comorbidity index. J Clin Epidemiol. 1994;47(11):1245-51.

9. Grymonprez M, De Backer TL, Bertels X, Steurbaut S, Lahousse L. Long-term comparative effectiveness and safety of dabigatran, rivaroxaban, apixaban and edoxaban in patients with atrial fibrillation: A nationwide cohort study. Front Pharmacol. 2023;14:1125576.

10. Yang CC, Fong Y, Lin LC, et al. The age-adjusted Charlson comorbidity index is a better predictor of survival in operated lung cancer patients than the Charlson and Elixhauser comorbidity indices. Eur J Cardiothorac Surg. 2018;53(1):235-40.

11. Kundi H, Coskun N, Yesiltepe M. Association of entirely claims-based frailty indices with long-term outcomes in patients with acute myocardial infarction, heart failure, or pneumonia: a nationwide cohort study in Turkey. The Lancet Regional Health -Europe. 2021;10:100183.

12. Segal JB, Chang HY, Du Y, Walston JD, Carlson MC, Varadhan R. Development of a Claims-based Frailty Indicator Anchored to a Well-established Frailty Phenotype. Med Care. 2017;55(7):716-22.

13. Segal JB, Huang J, Roth DL, Varadhan R. External validation of the claims-based frailty index in the national health and aging trends study cohort. Am J Epidemiol. 2017;186(6):745-7.

14. Grymonprez M, Petrovic M, De Backer TL, Steurbaut S, Lahousse L. Impact of frailty on the effectiveness and safety of non-vitamin K antagonist oral anticoagulants (NOACs) in patients with atrial fibrillation: a nationwide cohort study. European Heart Journal - Quality of Care and Clinical Outcomes. 2024;10(1):55-65.

15. Le Pogam M-A, Seematter-Bagnoud L, Niemi T, et al. Development and validation of a knowledge-based score to predict Fried's frailty phenotype across multiple settings using one-year hospital discharge data: The electronic frailty score. eClinicalMedicine. 2022;44:101260.

16. Austin PC, Stuart EA. Moving towards best practice when using inverse probability of treatment weighting (IPTW) using the propensity score to estimate causal treatment effects in observational studies. Stat Med. 2015;34(28):3661-79.
